# Characterization of antibiotic resistomes by reprogrammed bacteriophage-enabled functional metagenomics in clinical strains

**DOI:** 10.1038/s41564-023-01320-2

**Published:** 2023-02-09

**Authors:** Gábor Apjok, Mónika Számel, Chryso Christodoulou, Viktória Seregi, Bálint Márk Vásárhelyi, Tamás Stirling, Bálint Eszenyi, Tóbiás Sári, Fanni Vidovics, Erika Nagrand, Dorina Kovács, Petra Szili, Ildikó Ilona Lantos, Orsolya Méhi, Pramod K. Jangir, Róbert Herczeg, Bence Gálik, Péter Urbán, Attila Gyenesei, Gábor Draskovits, Ákos Nyerges, Gergely Fekete, László Bodai, Nóra Zsindely, Béla Dénes, Ido Yosef, Udi Qimron, Balázs Papp, Csaba Pál, Bálint Kintses

**Affiliations:** 1 Synthetic and System Biology Unit, Institute of Biochemistry, Biological Research Centre, National Laboratory of Biotechnology, Eötvös Loránd Research Network (ELKH), Szeged, Hungary; 2 Doctoral School of Biology, University of Szeged, Szeged, Hungary; 3 HCEMM-BRC Translational Microbiology Research Group, Szeged, Hungary; 4 Institute of Biochemistry, Biological Research Centre, National Laboratory for Health Security, Eötvös Loránd Research Network (ELKH), Szeged, Hungary; 5 Doctoral School of Multidisciplinary Medical Sciences, University of Szeged, Szeged, Hungary; 6 Bioinformatics Research Group, Genomics and Bioinformatics Core Facility, Szentágothai Research Centre, University of Pécs, Pécs, Hungary; 7 Department of Clinical Molecular Biology, Medical University of Bialystok, Bialystok, Poland; 8 Department of Biochemistry and Molecular Biology, Faculty of Science and Informatics, University of Szeged, Szeged, Hungary; 9 Department of Genetics, Faculty of Science and Informatics, University of Szeged, Szeged, Hungary; 10 Veterinary Diagnostic Directorate, National Food Chain Safety Office, Budapest, Hungary; 11 Department of Clinical Microbiology and Immunology, Sackler School of Medicine, Tel Aviv University, Tel Aviv, Israel; 12 HCEMM-BRC Metabolic Systems Biology Lab, Szeged, Hungary; 13 Present Address: Department of Zoology, University of Oxford, Oxford, UK

**Keywords:** Antimicrobial resistance, Genetic transduction, Pathogens, Genetic engineering

## Abstract

Functional metagenomics is a powerful experimental tool to identify antibiotic resistance genes (ARGs) in the environment, but the range of suitable host bacterial species is limited. This limitation affects both the scope of the identified ARGs and the interpretation of their clinical relevance. Here we present a functional metagenomics pipeline called Reprogrammed Bacteriophage Particle Assisted Multi-species Functional Metagenomics (DEEPMINE). This approach combines and improves the use of T7 bacteriophage with exchanged tail fibres and targeted mutagenesis to expand phage host-specificity and efficiency for functional metagenomics. These modified phage particles were used to introduce large metagenomic plasmid libraries into clinically relevant bacterial pathogens. By screening for ARGs in soil and gut microbiomes and clinical genomes against 13 antibiotics, we demonstrate that this approach substantially expands the list of identified ARGs. Many ARGs have species-specific effects on resistance; they provide a high level of resistance in one bacterial species but yield very limited resistance in a related species. Finally, we identified mobile ARGs against antibiotics that are currently under clinical development or have recently been approved. Overall, DEEPMINE expands the functional metagenomics toolbox for studying microbial communities.

## Abstract (web summary)

Application of reprogrammed bacteriophage to functional metagenomics in clinically relevant bacterial strains improves identification of antibiotic resistance genes, including those against recently developed or approved antibiotics.

## Main

Metagenomics allows the exhaustive analysis of microbial communities, including species that cannot be cultivated in laboratory conditions. By extracting genomic data from environmental samples, researchers gain knowledge on the species compositions and functionality of the microbiome in a range of natural environments^1^. In particular, functional metagenomics is devoted to screening metagenomic DNA for the presence of genes that encode specific molecular functions^2–4^. Cloning and expressing fragmented metagenomic DNA in a bacterial host can reveal previously undescribed proteins. Applications of functional metagenomics include the identification of enzymes, exploring bioactive agents and screening for antibiotic resistance genes residing in the environment^5–8^. The libraries typically contain millions of DNA fragments, corresponding to a total coverage of 5–100 Gb, the size of thousands of bacterial genomes^7,9,10^.

Although functional metagenomics can potentially be useful for several research areas, in its present form the methodology is far from perfect, limiting its applicability. Given the enormous size of the plasmid libraries, efficient introduction of these libraries into a bacterial host is of central importance. However, this process—typically by electroporation, conjugation or conventional bacteriophage transduction—is cumbersome and is only efficient for a limited range of laboratory strains^11,12^. This limitation has far-reaching consequences on the applicability of functional metagenomic screens and the generality of conclusions that can be drawn^13,14^. For example, it hinders screening for biotechnologically or clinically relevant genes that are functional in only specific bacterial species ^12,15,16^. In particular, most metagenomic screens for antibiotic resistance genes (ARGs) rely heavily on the use of laboratory strains of *Escherichia coli* as bacterial hosts^5,17,18^. Therefore, ARGs that do not provide resistance in these strains but do so in other clinically relevant pathogens remain undetectable. Indeed, previous studies indicate that the impact of antibiotic resistance mutations on resistance phenotypes depends on the bacterial host’s genetic background^19^. Additionally, metagenomic screens in multiple host bacteria could provide valuable information on interspecies functional compatibility and mobility of ARGs^20^.

**Fig. 1:**
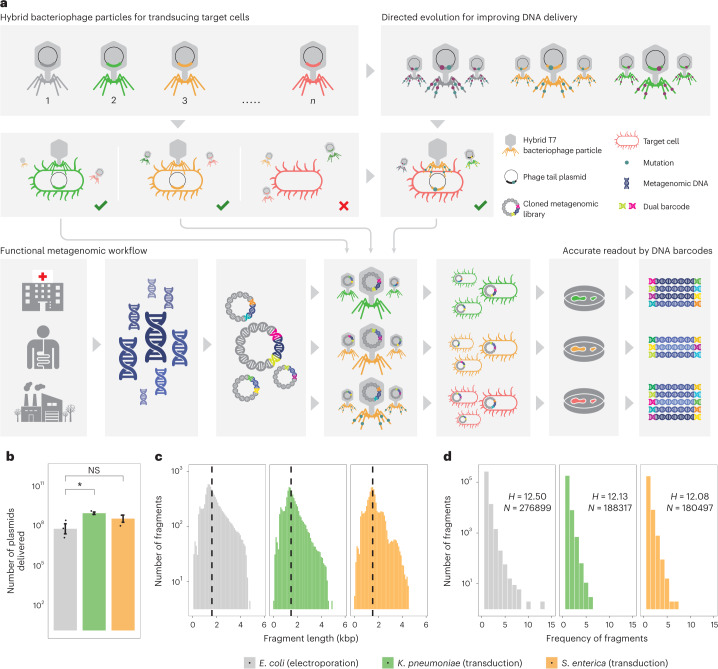
Functional metagenomics by reprogrammed bacteriophage particles. **a**, Schematic overview of DEEPMINE. DEEPMINE employs hybrid T7 bacteriophage transducing particles and directed evolution to alter phage host-specificity and efficiency for functional metagenomics in target clinical strains. Environmental DNA in the form of metagenomic plasmid library is then packaged into these bacteriophage particles and transduced into the hosts of interest. Comparative analysis of screening hits is enabled by a dual-barcoded PCR-free DNA fragment sequencing pipeline. **b**, Functional metagenomic library transduction by specific hybrid T7 bacteriophage particles is at least as efficient as electroporation (electroporation into *E. coli* vs transduction into *K. pneumoniae P* = 0.010545, two-sample one-sided *t*-test, *n* = 3 biologically independent experiments; electroporation into *E. coli* vs transduction into *S. enterica P* = 0.15, two-sample one-sided *t*-test, *n* = 3 biologically independent experiments; Supplementary Table 2). Mean ± s.e.m. **c**,**d**, Delivered metagenomic DNA fragment lengths (**c**) and diversities (**d**), determined by using PCR-free long-read deep sequencing right after electroporation and transduction (Methods). Dashed lines represent the average size of the DNA fragments. Shannon alpha diversity indices (*H*) were calculated on the basis of the frequency of fragments with identical sequences in the libraries (see Methods, n= 276,899, *n* = 188,317 and *n* = 180,497 for *E. coli*, *K. pneumoniae* and *S. enterica*, respectively; Supplementary Table 3). Please note that only a fraction of the delivered libraries was sequenced.

In this paper, we present Reprogramme**d** Bact**e**riophag**e P**article Assisted **M**ult**i**-species Fu**n**ctional M**e**tagenomics (DEEPMINE), which provides a solution to these problems (Fig. 1a). DEEPMINE is based on a previous work that aimed to extend the host range of T7 phage particles for DNA transduction by exchanging the tails between different types of bacteriophages^21^. DEEPMINE employs such modified bacteriophage transducing particles to deliver large metagenomic plasmid libraries into a range of bacterial species. Additionally, we applied directed laboratory evolution to increase the efficiency of such library delivery^22^. Using this approach, we performed metagenomic screens in clinically relevant bacterial pathogens from the Enterobacteriaceae family. We identified several previously unreported ARGs with species-specific effects on antibiotic susceptibility. Additionally, we studied a set of antibiotics that have only recently been approved for clinical use or are in late-stage clinical development, and show that these new antibiotics are just as prone to resistance formation as old antibiotics after decades of clinical use (Extended Data Table 1).

**Table 1:** ARGs identified against ‘recent’ antibiotics

Apramycin sulfate	Delafloxacin	Eravacycline	Omadacycline	Gepotidacin	Sulopenem	Ceftobiprole
AAC(3)-XI	ramA (2)^a^	ramA (2)	ramA (2)^a^	ramA (2)^a^	VIM-1^a^	CblA-1 (2)
PmpM	QnrB46	tet(X)^a^	adeS	cdeA (2)	NDM-1^a^	CcrA
AAC(3)-Ivb (8)	mfpA	tet(X5)^a^	marA (3)	evgS	AIM-1 (4)	cepA
aadS	QnrA1	marA (4)	mepA (2)	hmrM (3)	BAT-1	cfiA1
		Txr	tet(37)	marA	cfiA1	cmeB
			tet(X)^a^	mdfA	LMB-1 (2)	CTX-M-14^a^
			Txr	mdtK (3)	marA	DHA-7^a^
				mdtM (2)	mecR1	LMB-1 (2)
				PmpM (5)	NmcR^a^	mecR1
				QnrB46	OXA-198 (2)	mexB
					OXA-2	NDM-1^a^
					OXA-21^a^	OXA-198 (4)
					OXA-53	OXA-2
						OXA-209
						OXA-21^a^
						OXA-395
						OXA-444
						OXA-486
						OXA-53
						ramA^a^
						SHV-12^a^
						TLA-1
						TLA-2
						VIM-1^a^

Numbers in brackets show the number of detected ARG clusters (with 95% sequence identity threshold). ^a^Horizontally transferred (see Methods). Data are available in Supplementary Table 7.

## Results

### DNA library delivery by reprogrammed bacteriophage particles

We first tested whether hybrid T7 bacteriophage particles with exchanged tail proteins are suitable tools to deliver functional metagenomic plasmid libraries into bacterial cultures. In brief, we created metagenomic libraries to obtain environmental and clinical resistomes^23^, including (1) river sediment and soil samples from seven antibiotic polluted industrial sites in the close vicinity of antibiotic production plants in India (that is, anthropogenic soil microbiome)^24,25^, (2) feacal samples from 10 European individuals who had not taken any antibiotics for at least 1 yr before sample donation (that is, gut microbiome) and (3) samples from a pool of 68 multi-drug resistant bacteria isolated in healthcare facilities or obtained from strain collections (that is, clinical microbiome; see Methods, Fig. 1a and Supplementary Table 1).

DNA fragments ranging from 1.5 to 5 kb in size were shotgun cloned into a low-copy cloning plasmid capable of replication in selected orders of the class Gammaproteobacteria^26^ (see Methods). The plasmid DNA carries a packaging signal sequence that allows translocation of the plasmid into the T7 bacteriophage independent of the T7 genome (Fig. 1a). Each constructed library contained 3–5 million DNA fragments, corresponding to a total coverage of 25 Gb (that is, the size of ~5,000 bacterial genomes). The resulting plasmid libraries were packaged in two previously characterized hybrid T7 phage particles that display tail fibre proteins from *Salmonella* phage ΦSG-JL2 and *Klebsiella* phage K11^21^. The three metagenomic libraries were transduced into *Salmonella enterica* subsp. *enterica* serovar *Typhimurium* str. LT2 and *K. pneumoniae* NCTC 9131, both of which are known bacterial targets of these two hybrid T7 bacteriophage particles^21^. In parallel, we electroporated the libraries in the model bacterium *E. coli* K12 (Methods). Finally, we analysed whether transduction by T7 phage particles introduces any bias into the size and composition of the libraries (Methods).

**Extended Data Fig. 1:**
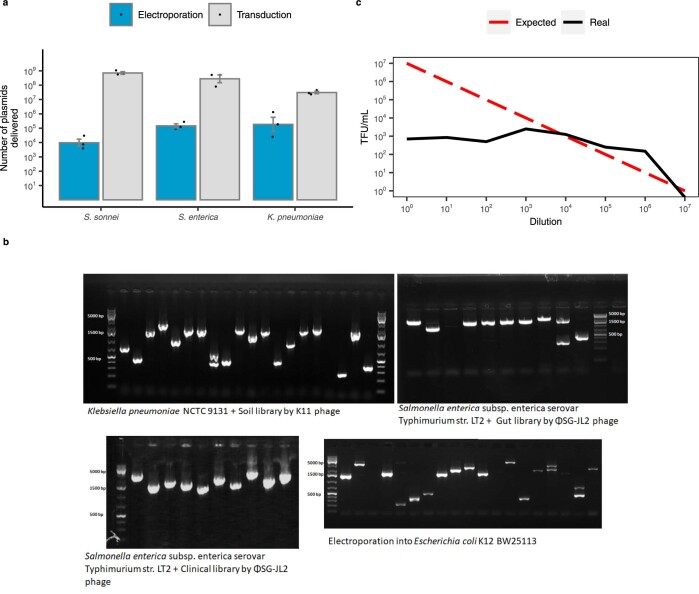
Characterization of functional metagenomic library delivery by hybrid T7 bacteriophage transducing particles into the target clinical strains. **a**, Comparison of electroporation and transduction efficiencies. The figure shows that the maximum number of plasmids delivered to the hosts is at least two orders of magnitude higher by transduction than by electroporation in all 3 pathogenic host species (Centre and error bars represent mean and standard error (*n*= 3 biologically independent experiments)). Data available in Supplementary Table 2. **b**, PCR amplified metagenomic inserts from transduced cells. Following transduction and electroporation, the metagenomic DNA fragments were PCR amplified by using plasmid specific primers at both sides of the metagenomic DNA fragment and subsequently sequenced by capillary sequencing. This experiment differentiates monoclonal cells (single PCR product and DNA sequence) from those that were co-transduced by two or more plasmids (double bands on the gel and mixed signal in the capillary sequence). PCR was repeated in case of each host-library pair with similar results. **c**, During the generation of transducing bacteriophage particles, a large portion of phages remains replicative and kills the bacterial cells used for phage generation. Therefore, with the increasing phage concentration, transduction efficiency is not growing as would be expected, but declines. The figure shows the transduction efficiency of the T7 transducing phage particle harboring T7 phage tail (black line) on *Shigella sonnei* HNCMB 25021 at different dilutions (see Methods). Red dashed line shows the expected increase in transduction efficiency without any detectable killing effect of replicative phages. Data available in Supplementary Table 2. Source data

Strikingly, both the ΦSG-JL2 and K11 tail-displaying hybrid T7 bacteriophage particles delivered the plasmid libraries into its targeted bacterial strain at least as efficiently as electroporation does into the laboratory *E. coli* model strain (Fig. 1b and Supplementary Table 2). In particular, the maximum number of plasmids delivered to the host bacteria were at least two orders of magnitude higher by transduction than by electroporation (Extended Data Fig. 1a and Supplementary Table 2).

Additionally, long-read deep sequencing shows that both the average DNA fragment sizes and the fragment diversities of the libraries delivered by T7 phage particles are comparable to that of the library delivered by electroporation into *E. coli* (Fig. 1c,d and Supplementary Table 3). This indicates that transduction by reprogrammed bacteriophage particles has no serious distorting effect on the size and diversity of the delivered metagenomic libraries. Finally, we sequenced the plasmid content of 38 isolated individual bacterial clones after phage transduction. Reassuringly, co-transduction of two plasmids into the same cell, a phenomenon that results in false positive hitchhiker hits in a screening campaign, was detected in only 5% of the cells, while co-transformation of two plasmids into the same cell by electroporation occurred in 10% of the cells (Extended Data Fig. 1b). Overall, these results indicate that certain T7 transducing bacteriophage particles with exchanged tail fibres are suitable delivery vehicles for functional metagenomics.

### Directed evolution optimizes DNA library delivery

Our next goal was to generalize our approach for the involvement of additional bacterial pathogen species. Transduction efficiencies of most hybrid phage particles are well below the threshold (>10^7^ transductants per ml) required for the delivery of entire functional metagenomic libraries into the target bacterial cells^21^. Moreover, the delivery of such libraries requires the use of high concentrations of the transducing phage particles. In such cases, replicative phage contamination, a common issue of transducing bacteriophage particle generation^27^, kills a large fraction of the target cells (Extended Data Fig. 1c).

**Fig. 2:**
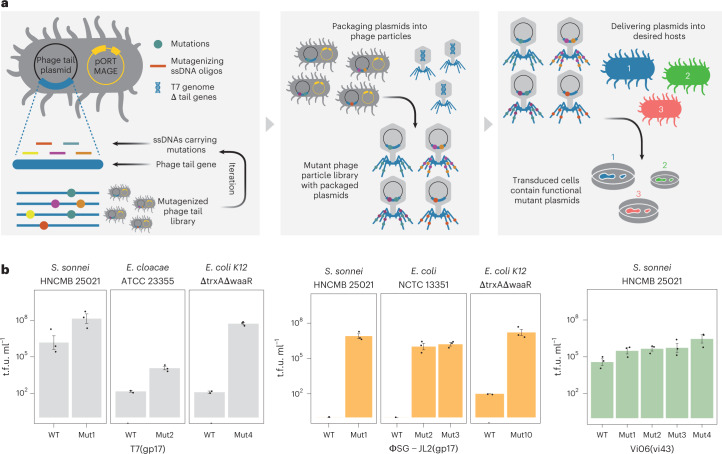
Directed evolution optimizes functional metagenomic library delivery. **a**, Schematic overview of the directed evolution experiment consisting of the following steps. (1) Phage tail mutagenesis in *E. coli* using DIvERGE. DIvERGE is a recombineering technique that incorporates soft-randomized single-stranded (ss) DNA oligonucleotides into multiple target sites (Methods). Phage tails are encoded on packageable plasmids. (2) Infecting the *E. coli* with T7 lacking the tail genes (T7Δ(gp11-12-17)). This step generates mutated phage particles, each containing the cognate mutant phage-tail-encoding plasmid. (3) Selection of phage tail variants that inject DNA into the selected target cells (1–3) with improved efficiency. The selection pressure is exerted by an antibiotic against which an antibiotic selection marker is encoded on the plasmid. **b**, Transduction efficiencies (t.f.u. ml^−1^) of the most efficient mutant tail fibres as compared to the parental WT tails. The target cells are *Enterobacter cloacae* ATCC 23355, *Shigella sonnei* HNCMB 25021 and *E. coli* NCTC 13351 as well as the phage-resistant *E. coli* model strain (BW25113ΔtrxAΔwaaR). Mean ± s.e.m. (*n* = 3 biologically independent experiments). Data are available in Supplementary Table 4.

To overcome these two problems, we set up a directed evolution experiment to genetically modify the tail fibre regions in the T7 phage particles. Specifically, we aimed to select for point mutations in the host-range-determining regions (HRDRs) of the phage tail fibres that alter host specificity^28,29^. To this end, we first selected three tail fibres (*Escherichia* phage T7, *Salmonella* phage ΦSG-JL2 and *Salmonella* phage Vi06) with especially broad host ranges^21^. Then, we identified potential HRDRs in the tail fibre gene gp17 of *Salmonella* phage ΦSG-JL2 and vi06_43 of *Salmonella* phage Vi06. The identification was based on sequence homology to four HRDRs in the receptor binding domain (RBD) of the well-characterized T7 and T3 phage tail fibre gene gp17 (Methods and Supplementary Table 4)^28,29^. Next, we introduced randomly distributed mutations within and in the vicinity of the HRDRs of tail fibre genes derived from ΦSG-JL2, Vi06 and T7 phages using a high-frequency site-directed mutagenesis method called DIvERGE (Fig. 2a and Methods)^22^. Compared with other mutagenesis protocols, DIvERGE has the advantage of introducing random mutations along multiple DNA sites simultaneously, and can cover relatively long DNA segments, potentially beyond the predicted HRDRs^22^.

Using a transduction optimization protocol^21^, we next selected phage tail variants with an improved capacity to deliver plasmid libraries into representatives of three pathogenic bacterial species: *Enterobacter cloacae* ATCC 23355, *Shigella sonnei* HNCMB 25021 and *E. coli* NCTC 13351 (Methods, Fig. 2b and Supplementary Table 4). Simultaneously, as a positive control, we selected the T7 phage tail library with the same protocol in the presence of a phage-resistant *E. coli* model strain (BW25113ΔtrxAΔwaaR) with deficient cell wall-embedded lipopolysaccharide receptors of T7-like phages^30,31^.

**Extended Data Fig. 2:**
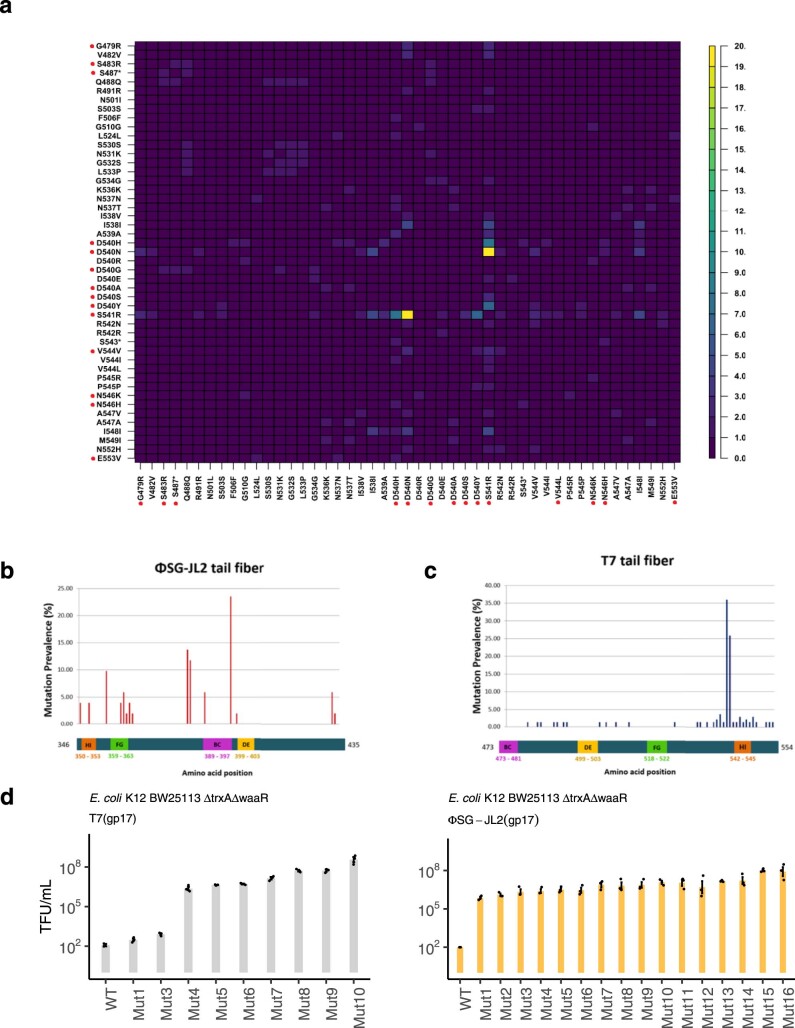
Distributions and transduction efficiencies of the most enriched mutations in the T7 and the ΦSG-JL2 tail fibre displaying hybrid T7 bacteriophage particles when selected on *E. coli* ΔwaaR model strain. **a**, The mutant T7 gp17 HRDRs usually carry specific combinations of mutations, 28% of which have been described as adaptive mutations previously. Heatmap representing the number of cases a mutation occurs in the 50 sequenced T7 phage tail HRDRs. Adaptive mutations according to (Huss et al.^28^) are indicated with a red dot. The frequent combination of specific adaptive mutations indicates the potential of DIvERGE to find host-specificity altering mutations with high efficiency. Data available in Supplementary Table 4. **b,c**, Distribution of detected mutations across the mutagenized phage tail fibre genes Escherichia phage T7 gp17 and *Salmonella* phage ΦSG-JL2 gp17. Predicted HRDRs are distinguished via colorized regions as in (Yehl et al.^29^) with the T3 bacteriophage. **d**, Transduction efficiencies of the mutant T7 (grey) and ΦSG-JL2 (yellow) phage tails as compared to their wild type counterparts with *E. coli* K12 BW25113 ΔtrxAΔwaaR LPS deficient strain. Y axis shows the number of transduced cells in 1 mL. Centre and error bars represent mean and standard error (*n* = 3 biologically independent experiments). Note that we did not observe any enriched mutant *Salmonella* phage Vi06 gp43, indicating that *Salmonella* phage Vi06 tail fibre binds to a cell surface receptor other than LPS. Data available in Supplementary Table 4. Source data

**Extended Data Fig. 3:**
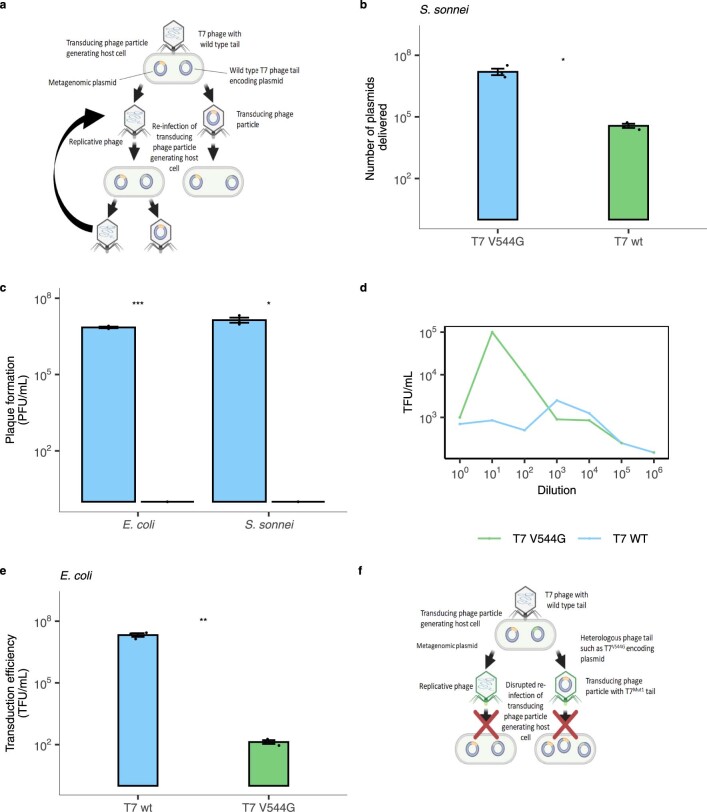
The effect of T7 gp17^V544G^ mutation on replicative phage contamination and on the transduction of the metagenomic plasmid. **a**, Schematic representation of transducing phage particle generation with T7 gp17^WT^. During the first step of the process, the T7 bacteriophage lacking its tail fibre encoding genes in its genome but otherwise displaying the wild type T7 tail fibre infects the *E. coli BW25113* cell carrying the metagenomic plasmid and a phage tail expressing plasmid. The infection results in the production of phage particles carrying either the metagenomic plasmid (transducing phage particle) or the phage genome (replicative phage) according to Yosef et al. If the phage tail encoded by the phage tail expressing plasmid (and therefore, displayed on the generated T7 particles) can efficiently infect *E. coli*, the replicative phage continoulsy accumulates during the process, since the phage genome containing phage particle can initiate a new reproduction cycle**. b**, The number of metagenomic plasmids that are delivered in *Shigella sonnei* HNCMB 25021 by the T7 phage particles harboring gp17^WT^ (blue) or gp17^V544G^ (green) tail fibers (two-sample one-sided *t*-test, *P* = 0.01944. Centre and error bars represent mean and standard error, *n* = 3 biologically independent experiments). Data is available in Supplementary Table 5. **c**, Replicative phage contamination measured by plaque formation of the T7 transducing phage particles harboring the gp17^WT^ (blue) or gp17^V544G^ (green) tail fibers (see Methods). Plaque assay was carried out both with *E. coli* BW25113 and with *S. sonnei* HNCMB 25021 (Two-sample two-sided t-test, *P* = 0.000168 and *P* = 0.013476 when applied with *E. coli* and *S. sonnei*, respectively. Centre and error bars represent mean and standard error; *n* = 3 biologically independent experiments). Data is available in Supplementary Table 5. **d**, Replicative phage contamination measured by transduced *S. sonnei* HNCMB 25021 colony numbers with T7 phage particles harbouring the gp17^WT^ (blue) or gp17^V544G^ (green) tail fibers. Lower amount of replicative phage in the T7 gp17^V544G^ transducing particle sample is indicated by the increasing colony numbers even at high concentrations of the transducing particle. Notably, unlike in Supplementary Fig. 5c, replicative phage activity is detected at the highest concentration of the transducing particle in this experiment. (*n* = 2 biological replicates. Centre and error bars represent mean and standard error.) Data available in Supplementary Table 5. **e**, Transduction efficiencies of the T7 phage particles harboring gp17^WT^ (blue) or gp17^V544G^ (green) tail fibers in *E. coli* BW25113 (two-sample two-sided *t*-test, *P* = 0.00553, *n* = 3 biologically independent experiments. Centre and error bars represent mean and standard error). Data is available in Supplementary Table 5. **f**, Schematic representation of the assumed transducing phage particle generation scheme with T7 gp17^V544G^. The decreased transduction efficiency for *E. coli* BW25113 abolishes the reproduction of the replicative phage after the first infection cycle. Note that the first infection cycle is carried out by the T7 gp17^WT^. In sum, the inefficient infection of *E. coli* by the mutant phage tail results in a lower amount of replicative phages. Source data

**Extended Data Fig. 4:**
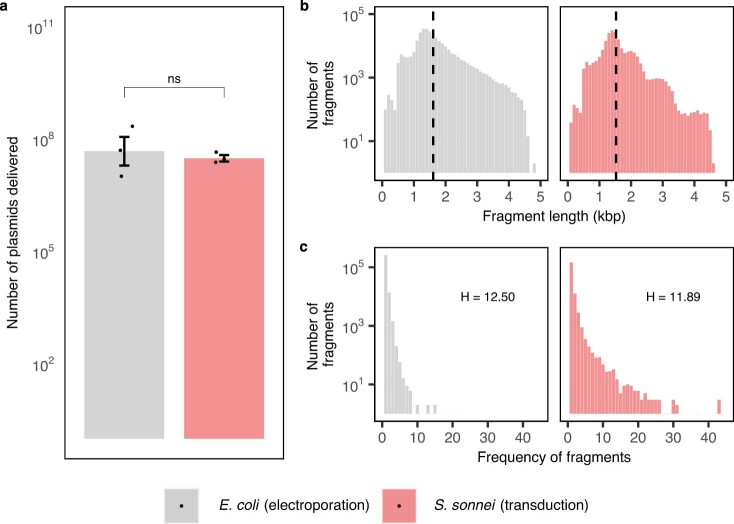
Functional metagenomic plasmid library delivery by gp17^V544G^ displaying T7 bacteriophage particles into *Shigella sonnei* HNCMB 25021. **a**, Functional metagenomic plasmid library delivery is as efficient by T7 gp17^V544G^ bacteriophage particles into *Shigella sonnei* HNCMB 25021 as by electroporation into *E. coli* BW25113 (*P* = 0.19769, two-sample one-sided *t*-test, *n* = 3 biologically independent experiments. Centre and error bars represent mean and standard error.) Data available in Supplementary Table 2. **b**,**c**, Delivered metagenomic DNA fragment lengths and diversities, respectively, determined by using long-read deep sequencing right after electroporation and transduction (Methods). Dashed lines represent the average size of the DNA fragments. Shannon alpha diversity indices (H) were calculated based on the frequency of fragments with identical sequences in the libraries (*n* = 276899, *n* = 162107, for *E. coli* and *S. sonnei*, respectively). Data available in Supplementary Table 3. Source data

As a result of directed evolution, DNA transduction efficiency was improved by one to seven orders of magnitude in all three pathogenic bacterial strains tested (Fig. 2b). With *Shigella sonnei* HNCMB 25021, the transduction efficiency reached the level suitable for the delivery of entire metagenomic plasmid libraries (Fig. 2b). In the case of the positive control ΔwaaR model strain, the most efficient mutant T7 gp17 HRDRs carry specific combinations of mutations, 28% of which have previously been described as adaptive mutations (Extended Data Fig. 2a–c). Overall, the adaptive mutations increased transduction efficiency (Fig. 2b, Extended Data Fig. 2d and Supplementary Table 4), and at least in one case (T7 gp17^V544G^ (Mut1 on Fig. 2b) with *Shigella sonnei* HNCMB 25021), it also minimized replicative phage contamination (for an explanation, see Extended Data Fig. 3 and Supplementary Table 5). Reassuringly, the transduction of the three metagenomic libraries into *Shigella sonnei* HNCMB 25021 by this T7 phage tail variant resulted in functional metagenomic libraries that are as large and diverse as the library achieved by electroporation in the *E. coli* K12 strain (Extended Data Fig. 4 and Supplementary Tables 2 and 3). Overall, we found that directed evolution of the phage tail improves the delivery of metagenomic libraries into previously untapped bacterial strains compared with the delivery of the same libraries by electroporation.

### Involving multiple pathogenic hosts expands the ARG repertoire

Our next goal was to improve sampling of the bacterial antibiotic resistome through functional metagenomics in multiple bacterial hosts. To this end, we screened the above-described three metagenomic libraries (soil, gut, clinical) in three pathogenic bacterial hosts (*Salmonella enterica* LT2, *K. pneumoniae* NCTC 9131 and *Shigella sonnei* HNCMB 25021) and in *E. coli* BW25113. The screens were performed on solid agar in the presence of one of 13 selected antibiotics covering five major antibiotic classes (Extended Data Table 1) at concentrations where the wild-type (WT) host strains are susceptible. The list includes six antibiotics (doxycycline (DOX), gentamicin (GEN), cefdinir (CFD), cefoxitin (CEF), meropenem (MER) and moxifloxacin (MOX)) with long clinical history (‘old’), and seven others (eravacycline (ERA), omadacycline (OMA), apramycin sulfate (APS), ceftobiprole (CEF), sulopenem (SUL), delafloxacin (DEL) and gepotidacin (GEP)) that have recently been introduced into the clinic (after 2017) or are currently in clinical development (‘recent’, as of April 2020, Extended Data Table 1). All studied antibiotics, including CEF^32^, have demonstrated activity against Gram-negative pathogens. Of note, APS has been used in veterinary medicine for over a decade but is currently under clinical trial to treat systemic Gram-negative infections in humans^33^.

**Fig. 3:**
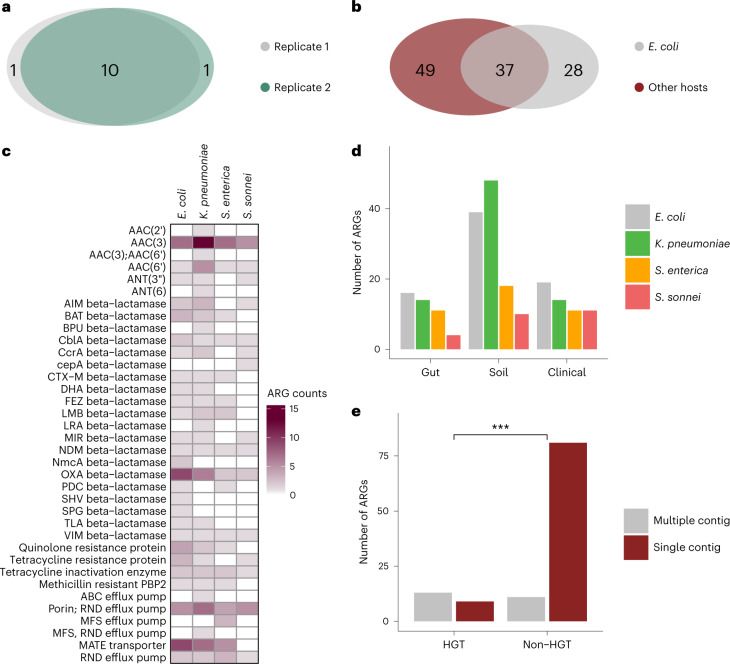
Involving multiple pathogenic hosts expands the ARG repertoire. **a**, Reproducibility of the pipeline. Venn diagram shows the number of ARGs detected in two biological replicate screens with *K. pneumoniae*. The intersection represents the ARGs isolated in both replicates, corresponding to an 83% reproducibility (Supplementary Table 6). **b**, Venn diagram showing the number of isolated ARGs using *E. coli* and the rest of the host species. When *E. coli* was used as the sole host, 43% of the total 114 ARGs remained undetected (Supplementary Table 6). **c**, Heat map showing gene families of the ARGs identified using the four host species. Colour code quantifies the number of identified ARGs belonging to the gene family (Supplementary Table 7). **d**, Number of ARGs identified in the four hosts across the three used resistomes (Supplementary Table 6). **e**, Number of mobile (depicted as HGT to denote the detection of involvement in horizontal gene transfer) and non-mobile (depicted as non-HGT to denote the lack of involvement in horizontal gene transfer) ARGs present on multiple and single contigs in the metagenomic libraries (two-sided Fisher’s exact test, *P* = 1.058 × 10^−5^, *n* = 114; Supplementary Table 6).

**Extended Data Fig. 5:**
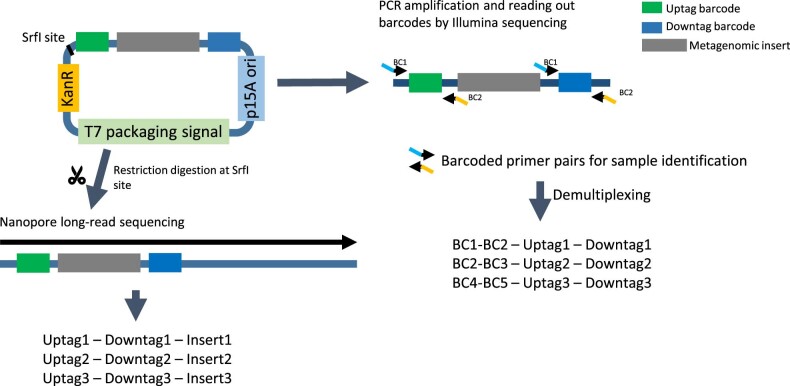
Schematic overview of the workflow used to sequence the metagenomic DNA fragments. The pipeline resembles a previously published workflow (Dual Barcoded Shotgun Expression Library Sequencing pipeline (Mutalik et al.^34^)) with a modification that avoids PCR amplification of resistance-conferring metagenomic DNA fragments, and therefore, preserves the original composition of the samples (Methods). The workflow consists of the following steps. First, all the functional metagenomic plasmids obtained from the screens were pooled and then linearized using SrfI restriction endonuclease. SrfI has an eight base-pair-long recognition sequence to minimize the digestion of the metagenomic insert. The linearized plasmids are then subjected to Nanopore long-read sequencing (Methods). Long-read sequencing identifies the metagenomic DNA fragment (insert) and the two 10 nucleotide long random barcodes pre-cloned up- and down-stream (Uptag and Downtag, respectively) of each metagenomic DNA fragment (Methods). Parallel, prior pooling the metagenomic plasmids from each screen, a multiplexed short-read deep-sequencing was applied to read out the plasmid-encoded unique barcodes on each side of the metagenomic fragments in each functional metagenomic screen. Specifically, the Uptag and Downtag sequences were PCR amplified with barcoded Illumina sequencing compatible primers (BC). Following illumina sequencing and demultiplexing of the samples using the BC barcodes, the Nanopore and Illumina datasets are combined to assign each plasmid (identified by the Up- and Downtags) to a screening batch that is a unique host, antibiotic and library combination.

The obtained resistance-conferring plasmids were pooled and sequenced with a modified dual-barcoded shotgun expression library sequencing pipeline (Extended Data Fig. 5 and Methods; see also ref. ^34^). The protocol avoids PCR amplification of resistance-conferring DNA fragments, thus preserving the original composition of the samples. By aligning the obtained DNA sequences to antibiotic resistance genes in relevant databases^35,36^, we found that 84% of the 571 fragments displayed sufficient sequence similarity (Methods) to known resistance genes (Supplementary Table 6). As many of the detected ARGs were isolated on several different DNA fragments, ARGs were clustered at 95% identity and coverage to reduce sequence redundancy in the dataset^37^. To quantify the reproducibility of the pipeline, we repeated the full protocol (one library delivery, screening and sequencing) with *K. pneumoniae*. Reassuringly, 83.3% of the ARGs were isolated in both biological replicates (Fig. 3a).

**Extended Data Fig. 6:**
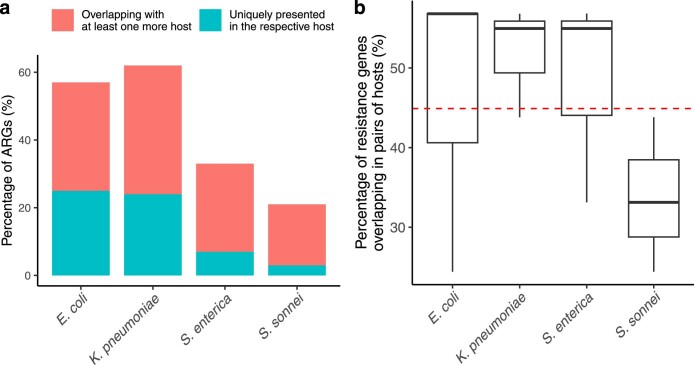
Percentage of ARGs identified in the four hosts. **a**, Percentage of ARGs identified in each of the four hosts uniquely (blue) and in overlap with at least one more host (red). *E. coli* identified only 57% of the 114 ARGs. **b**, On average, only 44% (labelled by a horizontal dashed line) of the detected ARGs overlap between pairs of species after accounting for the variability of the replicate screens (83%), that is dividing the percentage overlap between pairs of species by the percentage overlap between replicate screens (0.365/0.83). Data available in Source Data File 6. Boxplots show the median (center horizontal line), the first and third quartiles (bottom and top of box, respectively), with whiskers showing either the maximum (minimum) value or 1.5 times the interquartile range of the data; *n* = 80 for *E. coli*, *n* = 101 for *K. pneumoniae*, *n* = 56 for *S. enterica* and *n* = 37 for *S. sonnei*, where ‘*n*’ is the number of ARGs identified in the given host). Data is available in Supplementary Table 6.

**Extended Data Fig. 7:**
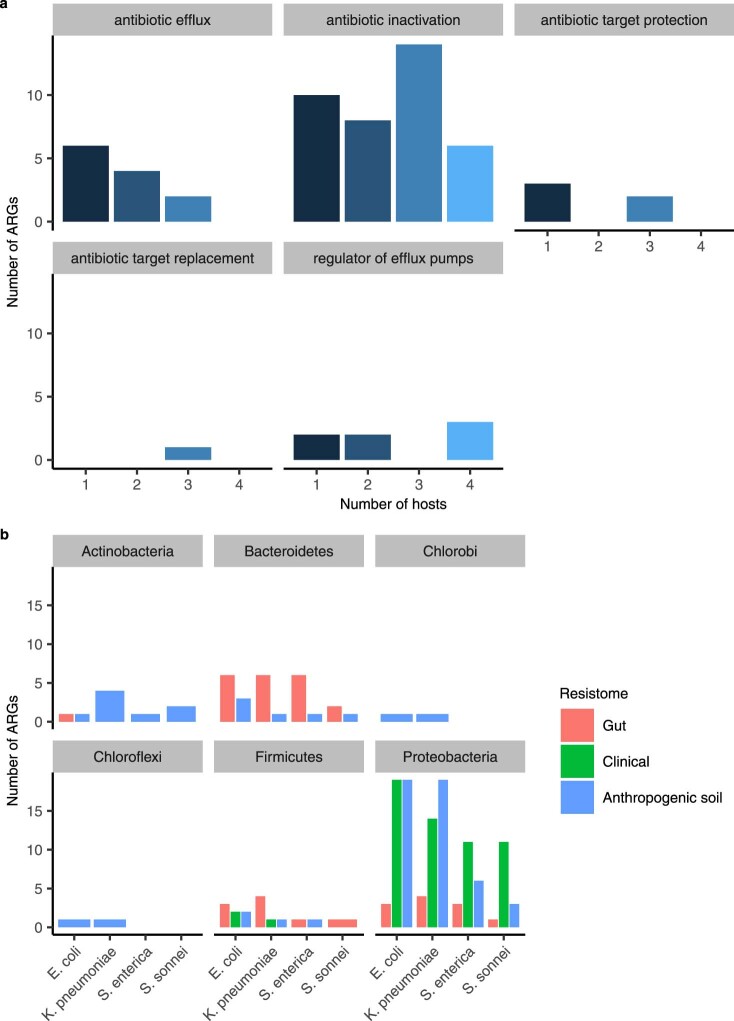
Phylogenetic origins and mechanistic categories of identified ARGs. **a**, The figure shows the number of ARGs originating from different phylogenetic groups and the distribution of each group across hosts and resistomes (*n* = 114). The majority of ARGs originated from Proteobacteria. Data available in Source Data File 7. **b**, The figure shows the number of ARGs identified in the different mechanistic categories and the functional compatibility of each category with multiple hosts (*n* = 114). The most frequent categories were antibiotic inactivation, antibiotic efflux and regulators of antibiotic efflux. Data available in Supplementary Table 7.

In total, 114 ARGs were detected, many of which were present in multiple DNA fragments (Supplementary Tables 6 and 7). The analysis also revealed substantial differences in the identified ARG repertoires across the four examined host bacterial species. In particular, when the analysis was restricted to *E. coli* as the bacterial host, 43% of the total 114 ARGs remained undetected (Fig. 3b–d and Extended Data Fig. 6). This indicates that DEEPMINE allows a more comprehensive sampling of the bacterial resistomes by the utilization of multiple host bacteria. Efflux pumps, their corresponding transcriptional regulators and antibiotic inactivating enzymes were common among the detected ARGs (Fig. 3c and Extended Data Fig. 7a). A substantial fraction of the ARGs isolated from the gut, soil and clinical microbiomes originated from Proteobacteria, which are phylogenetically close relatives of the host bacterial species in our screens (Extended Data Fig. 7b).

Then, we determined whether the ARGs detected in our screen are prone to horizontal gene transfer in nature. ARGs that have been mobilized in the past in human-associated environments may pose a higher health hazard as they have the potential to become widespread among human pathogens^38^. To investigate this issue, we generated a mobile gene catalogue on the basis of identification of nearly identical genes that are shared by distantly related bacterial genomes^37,39,40^. Specifically, we carried out the pairwise alignment of 2,794 genomes of phylogenetically diverse human-related bacterial species (Supplementary Table 8). This dataset was extended with a sequence database of 27,939 natural plasmids derived from diverse environments (ref. ^41^, Methods). ARGs carried by plasmids were especially likely to be transferred between bacterial species, with a 91% agreement between the two datasets on mobile ARGs (Supplementary Table 7). Remarkably, ARGs present in multiple DNA fragments in our screen were more frequently subjected to horizontal gene transfer in nature compared with ARGs that are only present in a single DNA fragment (Fig. 3e).

### Species-specific activity of ARGs across bacterial species

Next, we asked how the variation in the detected ARG repertoires across the four bacterial hosts can be explained. The first hypothesis was that certain ARGs remain undetected due to stochastic plasmid loss. This can happen during transduction of the metagenomic library into their new hosts or during the screening process. Alternatively, the transferred ARGs may not be functionally compatible with the physiology of all bacterial hosts^20^. Therefore, several ARGs provide resistance in specific bacterial species only. While the first hypothesis is certainly relevant, several lines of evidence indicate substantial differences in the resistance phenotype of ARGs across bacterial species.

**Fig. 4:**
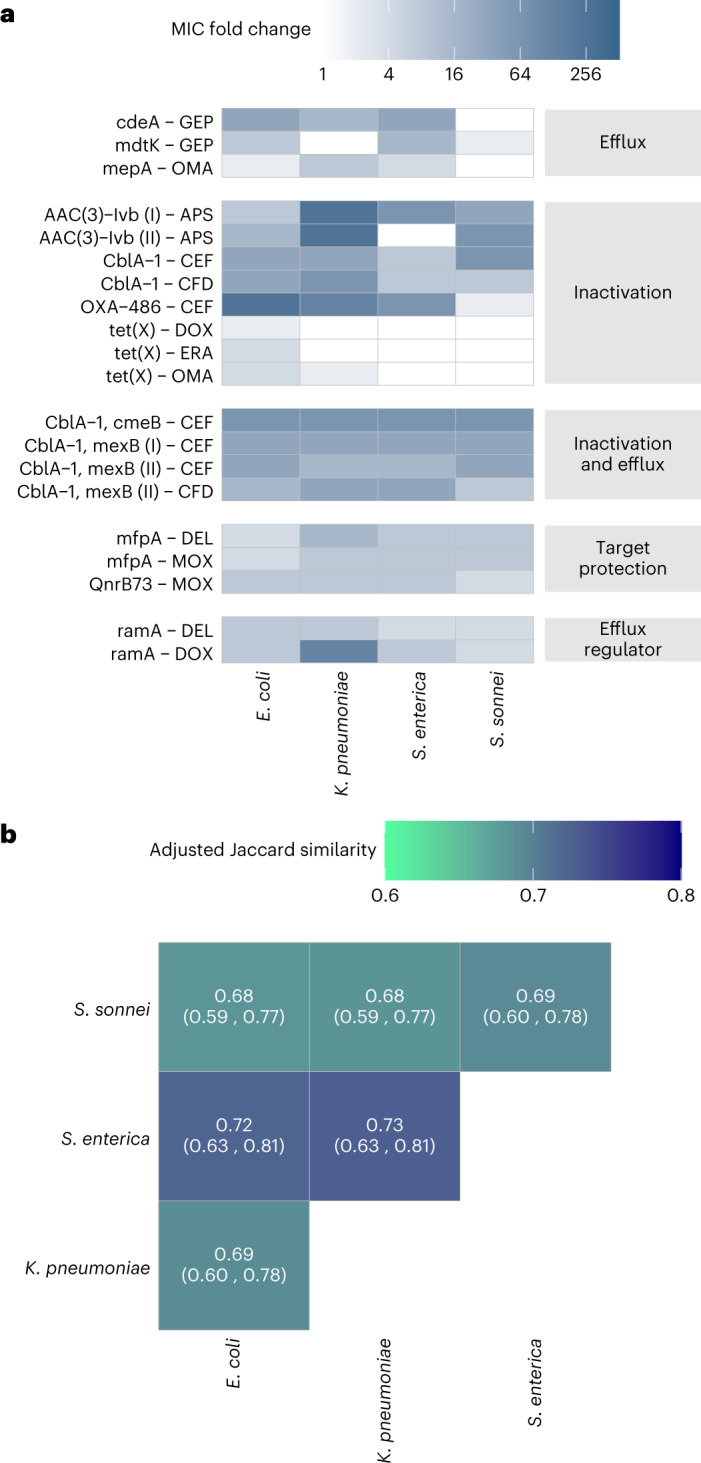
Species-specific activity of ARGs across bacterial species. **a**, Heat map showing substantial variation in the resistance levels provided by the 13 resistance-conferring DNA fragments in the four host species. Colour code quantifies MIC fold changes. White colour means no change in resistance level, using a twofold change in MIC as a cut-off. Data are available in Supplementary Table 9. **b**, Adjusted Jaccard similarity coefficients that represent the overlaps of functional ARG sets between pairs of host species after controlling for measurement noise (see Methods and Extended Data Fig. 8). Numbers in brackets represent 95% confidence intervals (Methods).

To test these hypotheses, we first examined how DNA fragments that provide antibiotic resistance in *E. coli* shape antibiotic susceptibility in the other three host bacterial species. We analysed a representative set of 13 resistance-conferring DNA fragments derived from our screens by measuring the levels of antibiotic resistance they provide across the bacterial hosts. As certain ARGs have been detected in multiple antibiotic screens, we studied 20 antibiotic–DNA fragment combinations in total (Fig. 4a). In seven out of the 20 studied cases, the DNA fragment provided no changes in resistance level in at least one of the three other bacterial species (using a twofold change in minimum inhibitory concentrations (MIC) as a cut-off). Therefore, on average, only 80% of the functional ARGs overlapped between the pairs of *E. coli* and the other three species. Additionally, we observed a substantial, up to 256-fold variation in the resistance level provided by the specific DNA fragments (Fig. 4a and Supplementary Table 9). Efflux pumps, transcriptional regulatory proteins and antibiotic-modifying enzymes alike displayed such major variation in resistance levels across the studied bacterial species (Fig. 4a).

**Extended Data Fig. 8:**
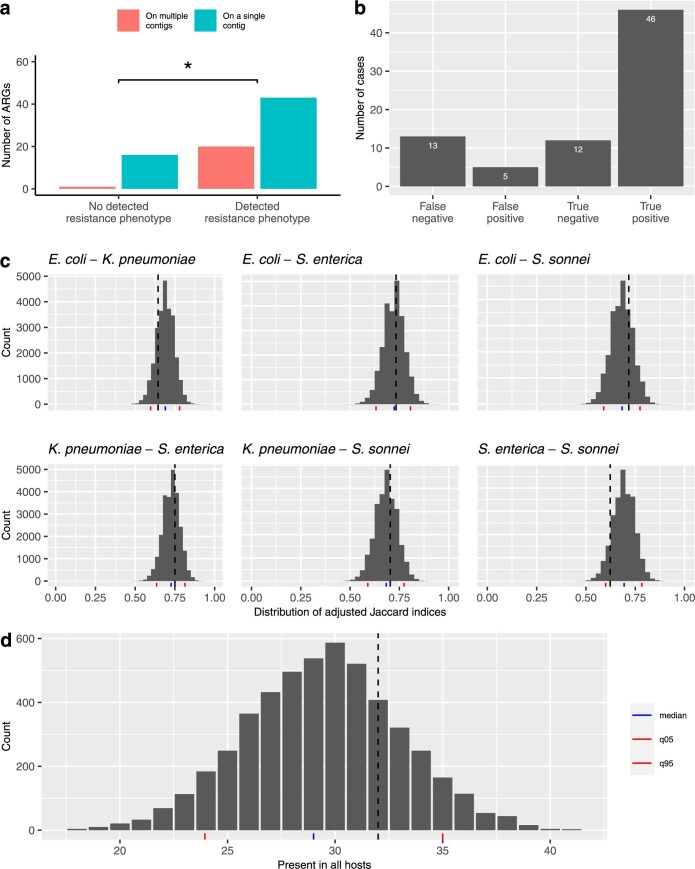
Re-investigation of all resistance-conferring DNA fragments from the metagenomic screens. **a**, A significantly higher portion of ARGs not being detected to provide a resistance phenotype in any species were present on a single resistance-conferring DNA fragment as compared to ARGs being detected to provide a resistance phenotype in at least one species (Two-tailed Fisher’s exact test, *P* = 0.032, *n* = 80, Supplementary Table 10). **b**, Estimated accuracy of the screen based on taking the MIC measurements as a gold standard dataset. Note that we excluded one ARG (QnrB73) from the MIC measurements, as re-introduction of this ARG into each of the four host bacterial species was not confirmed by sequencing of the plasmid library (Source Data File 9). Presence of resistance in the MIC dataset was defined as a more than two-fold change in relative MIC value. False negative hits are those ARGs that were not detected in the screen but showed a resistance phenotype in the MIC measurements. False positive hits are those that did not provide resistance in the MIC measurements but were detected to show a resistance phenotype in the screen. We assumed plasmid hitchhiking as a primary source of false positives. Data is available in Supplementary Table 9**c**, The distribution of adjusted Jaccard similarity coefficients that represent the overlaps of functional ARG sets between pairs of host species after controlling for measurement accuracy using a stochastic approach (Methods). Dashed line, blue line and red lines represent the measured Jaccard similarity coefficient for host species pairs, the median of the adjusted Jaccard similarity coefficients and the lower and upper bounds of the 95% confidence intervals, respectively. **d**, In total, only ~46% of the ARGs (~29 out of 63) are estimated to provide resistance in all four bacterial host species. Histogram shows the number of ARGs that are estimated to confer resistance in all four host species when taking into account the false positive and false negative rates of the screen by using a stochastic approach (see Methods). Dashed line, blue line and red lines represent the measured Jaccard similarity coefficient for host species pairs, the median of the adjusted Jaccard similarity coefficients and the lower and upper bounds of the 95% confidence intervals, respectively. (see Methods).

Finally, we re-investigated all resistance-conferring DNA fragments detected in the metagenomic screens. We pooled the corresponding plasmids and re-introduced the resulting pre-selected plasmid library into each of the four native bacterial host species. We subsequently performed new antibiotic selection screens with this library on solid agar, as previously described. To control for stochastic plasmid loss during transduction, we sequenced the new plasmid library before and after antibiotic selection. Of the ARGs, 70% (80 out of 114) were represented by at least one plasmid in all four bacterial host species after transduction, but before antibiotic selection (Supplementary Table 10). After antibiotic selection, 63 of these ARGs were detected to show antibacterial activity in at least one of the four bacterial host species (Supplementary Table 10). Notably, 16 out of the 17 ARGs lost during antibiotic selection were encoded by only a single resistance-conferring DNA fragment (Extended Data Fig. 8a). After adjusting the overlaps with the accuracy of the screen (Extended Data Fig. 8b), on average, 70% of the ARGs overlapped between pairs of species (Fig. 4b and Extended Data Fig. 8c). In total, only ~46% of the ARGs (~29 out of 63) provided resistance in all four bacterial host species (Extended Data Fig. 8d). Clearly, future work on larger metagenomic datasets should reveal the exact biochemical, cellular and phylogenetic features that shape the species-specificity profiles of ARGs.

Together, these results indicate that ARGs, when transferred to new bacterial hosts, frequently have species-specific effects on antibiotic susceptibility.

### Potential resistance to recently developed antibiotics

**Fig. 5:**
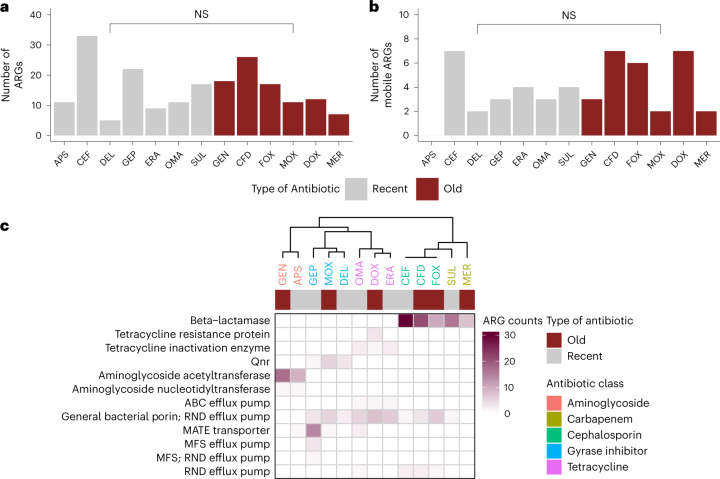
Potential resistance to recently developed antibiotics. **a**, The overall numbers of ARGs are statistically the same for ‘recent’ and ‘old’ antibiotics (two-sided Wilcoxon rank-sum test, *P* = 0.8051, *n* = 107 for ‘old’ and *n* = 114 for ‘recent’; Supplementary Table 7). **b**, The same is true for ARGs with established horizontal gene transfer events (*P* = 0.6106, two-sided Wilcoxon rank-sum test, *n* = 27 and 23 for ‘old’ and ‘recent’ antibiotics, respectively; Supplementary Table 7). **c**, Resistance mechanisms largely overlap between ‘old’ and ‘recent’ antibiotics belonging to the same drug classes. Heat map shows the clustering of the antibiotics based on the ARG profiles. Colour coding quantifies the number of detected ARGs that are grouped by mechanism. Data are available in Supplementary Table 7.

**Extended Data Fig. 9:**
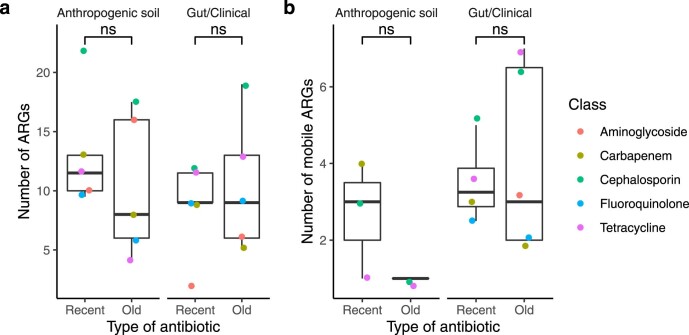
Number of ARGs and mobile ARGs identified in the different antibiotic classes. **a**, The overall number of ARGs are statistically the same for the old and new antibiotic groups, no matter which microbiomes were considered. (anthropogenic soil: *P* = 0.4377, human-associated (gut/clinical): *P* = 0.601, Welch Two-Sample two-sided *t*-test, *n* = 5 and *n* = 5 for new and old, where *'n'* represents the number of antibiotics, respectively; boxplots show the median (center horizontal line), the first and third quartiles (bottom and top of box, respectively), with whiskers showing either the maximum (minimum) value or 1.5 times the interquartile range of the data). **b**, The above results remained when the analysis was restricted to ARGs with established horizontal gene transfer events (anthropogenic soil: *P* = 0.1994, Welch Two-Sample two-sided *t*-test, *n* = 3 and *n*= 2 for new and old, where '*n*' represents the number of antibiotics, respectively; human-associated (gut/clinical): *P* = 0.6426, Welch Two-Sample *t*-test, *n* = 4 and *n* = 5, for new and old, respectively). Data is available in Supplementary Table 7.

Next, we estimated how prone the ‘recent’ antibiotics are to ARG mobilization compared to the ‘old’ antibiotics. We found that the overall numbers of ARGs are statistically the same for the two antibiotic groups (Fig. 5a, Table 1), regardless of the microbiomes that were considered (Extended Data Fig. 9a). Moreover, when the analysis was restricted to ARGs with established horizontal gene transfer events, the above results remained (Fig. 5b and Extended Data Fig. 9b). As expected, the resistance mechanisms largely overlap between ‘old’ and ‘recent’ antibiotics belonging to the same drug classes (Fig. 5c), suggesting that cross-resistance could be prevalent. CEF, a fifth-generation cephalosporin that has recently been approved for the treatment of hospital- and community-acquired pneumonia^42,43^ highlights this point. Both the overall frequency of ARGs (for example, *β*-lactamases) and the frequency of mobile ARGs were exceptionally high against CEF (Table 1), even when compared to those of ‘old’ *β*-lactam antibiotics with decades of clinical use (Fig. 5a–c). Indeed, extended-spectrum *β*-lactamases (ESBLs) generally hydrolyse ceftobiprole^44^, hence its clinical utility against Gram-negative multidrug-resistant pathogens producing such ESBLs is limited^45^.

A notable exception to this trend is APS, an antibiotic in clinical trial for application in humans. Only a single ARG was detected against this antibiotic in the gut resistome and none in the pooled collection of clinical isolates (Supplementary Table 7). However, in agreement with extensive use of APS in veterinary medicine for decades, multiple ARGs against APS were detected in the soil microbiome (Fig. 5c). The identified ARGs are mostly aminoglycoside acetyltransferases that are functionally compatible in multiple pathogenic hosts (Table 1, Supplementary Table 7 and Fig. 5c). This suggests that these genes can be of potential clinical risk in the future. In agreement with this expectation, one of these aminoglycoside acetyltransferases, *AAC(3)-IV*, has already been detected in APS-resistant clinical bacteria^46^. Overall, DEEPMINE could be a useful tool to predict ARGs currently only detectable in non-human-associated microbiomes with potential health implications.

## Discussion

In this work, we introduce DEEPMINE, an approach that broadens the range of host bacterial species applicable in functional metagenomics. Previous work showed that bacteriophage host range can be broadened by exchanging the tail fibre of the *E. coli* phage T7 or by generating random mutations in the T7 tail-fibre-encoding genes^21^. DEEPMINE employs such reprogrammed bacteriophage transducing particles with exchanged and/or mutagenized tail fibres to deliver large metagenomic plasmid libraries into a range of bacterial species (Fig. 1). The main advantage of DEEPMINE over existing techniques for functional metagenomics, such as electroporation or conjugation, is its higher efficiency. In particular, we found that DEEPMINE is more suitable for introducing small-insert (1.5 kb–5 kb) metagenomic plasmid libraries to the selected bacterial hosts than electroporation (Fig. 1 and Extended Data Fig. 1)^4,47^. While conjugation is frequently used to deliver libraries with large insert sizes (10 kb–40 kb) that typically contain 10^4^–10^5^ clones, it is very challenging to obtain more than 10^6^–10^7^ transconjugants with this technique^48,49^. On the other hand, a small-insert (1.5 kb–5 kb) metagenomic library such as used in this study usually requires more than >10^8^ plasmids to deliver libraries with sufficient coverage.

Using our approach, we performed 156 metagenomics screens with all possible combinations of 13 antibiotics, three metagenomic libraries (isolated from soil, gut and clinical microbiomes) and four related Enterobacteriaceae species. We demonstrate that by studying multiple host species, the bacterial resistome is substantially expanded; 43% of the non-overlapping ARGs remain undetected when only a single species (*E. coli*) was considered (Fig. 3). Accordingly, DEEPMINE allows the identification of ARGs that provide resistance only in specific clinically relevant pathogens. Indeed, we identified a large set of ARGs against recently developed antibiotics with potential to become future health risks (Fig. 5). On the basis of these results, we anticipate that DEEPMINE will be a useful tool to predict the future dissemination of ARGs for which there is a growing general interest^6,16,37,38,50^. However, the current limitation of DEEPMINE is that it takes considerable time and resources to engineer suitable phage particles to enable host bacteria of interest to be used for functional metagenomics.

In summary, our work provides a deeper insight into the forces that shape the mobile resistome. Future work should expand the metagenomic libraries involved to classify mobility and functional compatibility of the detected ARGs in a more comprehensive manner and test in a broader range of clinical isolates.

## Methods

This research complies with all relevant ethical regulations approved by the Human Investigation Review Board of Albert Szent-Györgyi Clinical Centre of the University of Szeged and the National Biodiversity Authority (NBA) of India. Permission for the faecal sample collection was obtained from the Human Investigation Review Board of Albert Szent-Györgyi Clinical Centre, University of Szeged (registered under 72/2019-SZTE). Volunteer participants were selected on the basis of strict criteria that (1) they did not take any antibiotics for at least one yr before sample donation and (2) they are in a good health. These requirements are standard in the field and secure a bias-free comparison of the antibiotic resistomes in the healthy human gut microbiome. Informed consent was obtained from all participants. Soil and river sediment sample collection from around the city of Hyderabad and Lucknow was approved by the National Biodiversity Authority (NBA), India (application number: NBA/Tech Appl/9/1822/17/18-19/3535). No statistical methods were used to pre-determine sample sizes, but our sample sizes are similar to those reported in previous publications^18,51,52^. Samples were not allocated to experimental groups. Samples for each individual experiment were handled by one person in charge. Data collection and analysis were not performed blind to the conditions of the experiments. No data were excluded from the analysis. Unless otherwise stated, when using a kit, we followed the manufacturer’s instructions.

### Plasmid construction for DEEPMINE

A custom plasmid was created from pZE21 expression vector (Supplementary Table 11) for compatibility with the T7 transduction and the sequencing pipelines. Specifically, the replication origin was switched from ColE1 to p15A, and the packaging signal of the T7 bacteriophage was introduced (enzymes and primers used are listed in Supplementary Table 11). Subsequently, the pZE21_p15A vector was amplified by PCR using a mixture of primers containing 10-nt-long random barcodes (Supplementary Table 11), followed by digestion and self-ligation.

### Sample collection and construction of metagenomic libraries

For the gut microbiome library, we collected faecal samples from 10 unrelated, healthy individuals with no history of taking antibiotics in the year before sample donation. For the anthropogenic soil microbiome, samples were collected from highly antibiotic-contaminated industrial areas in India^53^. Metagenomic DNA from the gut and soil samples was extracted using DNeasy PowerSoil kit (Qiagen, 47016). Genomic DNA of clinical bacterial isolates (Supplementary Table 1) was isolated using the Sigma GenElute bacterial genomic DNA kit (Sigma, NA2110-1KT).

From each sample, 40 µg of extracted DNA was digested with *Mlu*CI enzyme (NEB, R0538L) (10 min, 37 °C), followed by inactivation (20 min, 85 °C). The quantity of the *Mlu*CI enzyme was varied to obtain DNA in the target size range of 1–5 kbp. DNA was isolated with pulsed field gel electrophoresis (Sage Science, PB02901) with a 0.75% agarose gel cassette and low-voltage 1–6 kbp marker S1 cassette definition. The metagenomic DNA fragments were ligated into the pZE21_p15A plasmid at the *Eco*RI site using a 3:1 mass ratio of insert:vector. Pure ligation mixture was electroporated into 40 µl of either *E. coli* MegaX (Invitrogen, C640003) or *E. coli* 10G ELITE (Lucigen, 60080-2) cells. Following one h of incubation at 37 °C, transformants were plated onto 50 µg ml^−1^ kanamycin containing Luria Bertani (LB) agar plates in 10^1^×, 10^2^× and 10^3^× dilutions for colony forming unit determination. The rest of the recovered cells were grown overnight on LB agar plates supplemented with kanamycin. The next day, plasmids were isolated. Insert size distribution was estimated by PCR amplification of relevant plasmid regions from 10–20 randomly selected clones. The average insert size was determined to be 2–3 kbp.

### Transducing hybrid bacteriophage particle preparation

Transducing hybrid bacteriophage preparation was adapted from ref. ^21^. In brief, *E. coli BW25113* cells containing phage-tail-encoding plasmids (Supplementary Table 11) were grown to optical density (OD)_600nm_ ~0.7 (250 r.p.m. at 37 °C), then placed on ice for 15 min. Next, cultures were centrifuged (2,200 × *g*, 4 °C, 10 min), supernatant was discarded and the cells resuspended in the same amount of medium (LB or Terrific Broth (TB)). Afterwards, T7 bacteriophages lacking T7 fibre-encoding regions (T7∆(gp11-12-17)) were used to infect cells at multiplicity of infection (MOI) 2–3. Following 2 h of incubation (100 r.p.m., 37 °C), cells were treated with 2% chloroform and vortexed. The mixture was then centrifuged with the same parameters as above. Finally, the supernatant containing phage particles was collected.

### Measuring transduction efficiency

Transduction efficiencies were measured as previously described^21^. In brief, target bacterial cells were grown to OD_600_ ~0.5 (250 r.p.m. at 37 °C), followed by 15-min-long incubation on ice, during which dilutions of the transducing phage particles were prepared with tenfold dilution steps. Then, 50 µl of target cells were mixed with 50 µl of phage particles from each dilution. Plates were incubated at 37 °C at 180 r.p.m. for 1 h. Samples then were spotted on antibiotic-supplied agar plates. Transductant forming units per ml (t.f.u. ml^−1^) were calculated on the basis of colony counts.

### Assembly of transducing particles containing the metagenomic libraries

*E. coli* K12 BW25113 strain containing phage-tail-encoding plasmids were electroporated with 30 ng of each plasmid library in five parallels to achieve suitable colony numbers, then plated on antibiotic-containing LB agar plates and grown overnight. Following growth, cells were stored in 20% glycerol at −80 °C. Next, frozen cells containing the library were grown in 40 ml LB supplemented with kanamycin 50 and streptomycin 100 by shaking at 230 r.p.m. at 37 °C until OD_600_ 0.7. Cells were cooled down on ice, centrifuged at 2,000 × *g* (4 °C, 10 min) and resuspended in LB medium. Then, the T7∆(gp11-12-17) bacteriophage was used to infect cells at MOI 2–3. Following 2 h of incubation (100 r.p.m. at 37 °C), cells were treated with 2% chloroform and vortexed. The mixture was then centrifuged and supernatant was collected.

### Delivery of the metagenomic libraries by transducing phage particles and by electroporation

Overnight cultures of the corresponding bacterial strains were diluted to OD_600_ 0.1 in 50 ml LB medium to grow at 230 r.p.m. at 37 °C until OD_600_ 0.5. Next, we added 20 ml of library containing transducing particles to the cells, followed by one h incubation at the same parameters. Next, cells were centrifuged at 2,200 × *g* for 10 min at 4 °C, resuspended in 1–5 ml LB medium, plated on LB + kanamycin 50 and grown overnight. The next day, cells were collected and stored with glycerol at −80 °C. Of each library, 50 ng was electroporated into *E. coli* K12 BW25113 in five parallels. Cells were recovered in SOC medium for one h at 37 °C and plated on LB + kanamycin50 plates and grown overnight. The next day, cells were collected and stored in 20% glycerol at −80 °C.

### Phage tail mutagenesis

To locate the HRDRs of the tail fibre genes, we used pairwise sequence alignment, where the recently identified HRDRs of gp17 of T3 coliphage^29^ were aligned to the tail fibre sequences of *Escherichia* phage T7 gp17, *Salmonella* phage ΦSG-JL2 gp17 and *Salmonella* phage Vi06 gp43. The determined sites and the proximal regions were then subjected to targeted mutagenesis by DIvERGE^22^, a technique based on the targeted incorporation of mutational load carrying 90-mer oligos. In brief, *E. coli BW25113* cells carrying the phage-tail-encoding plasmid to be mutated and the plasmid mediating the mutagenesis^22^ were grown to ~OD_600_ 0.3–0.4 in TB (250 r.p.m. at 37 °C) supplied with appropriate antibiotics. Next, m-toluic acid was added (1 mM final concentration) to induce gene expression and after one h incubation, cells were transferred to ice for 15 min. Cell culture was made electrocompetent by repeated washing and centrifuging (2,200 × *g*, 4 °C, 10 min, three times), then electroporated with 2.5 µM oligos (Supplementary Table 11). Following recovery in TB (250 r.p.m., 37 °C, one h), cells were transferred to 19 ml TB supplied with appropriate antibiotics and left to grow overnight. Mutagenesis cycle was repeated if it was deemed necessary.

### Selection of mutant phage tails with improved transduction efficiency

To select for tail mutants with improved delivery capacity, we applied a transduction optimization protocol. In brief, we chose three pathogenic bacterial strains (*Enterobacter cloacae* ATCC 23355, *Shigella sonnei* HNCMB 25021 and *E. coli* NCTC 13351) based on initial weak T7 bacteriophage infectivity. These target bacterial cells were grown to ~OD_600_ 0.5 (250 r.p.m. at 37 °C) in LB, cells were placed on ice for 15 min, mixed with 2 ml of phage particles in a 1:1 volume ratio, and incubated at 37 °C and 100 r.p.m. for one h. The mixture was then plated and placed at 37 °C to grow overnight. The same protocol was carried out with non-mutagenized wild-type phage-tail-carrying particles. Colonies were pooled the next day and plasmid DNA was isolated using GeneJET plasmid miniprep kit (Thermo Fisher), then further purified using DNA Clean and Concentrator-5 (Zymo Research kit, D4004). Of the plasmids, 100 ng were electroporated into *E. coli BW25113* cells. After recovery, cells were supplied with appropriate antibiotics, spread onto agar plates after one h of incubation and left to grow overnight. The following day, the cells were pooled in 4 ml LB, 250 µl were transferred into 40 ml TB supplied with appropriate antibiotics and grown to ~OD_600_ 0.7 (250 r.p.m. at 37 °C). After growth, cells were placed on ice for 15 min, centrifuged (2,200 × *g*, 4 °C, 10 min) and resuspended. Next, cell cultures were infected with T7∆(gp11-12-17) bacteriophages. After two h (100 r.p.m. at 37 °C), cells were treated with 2% chloroform and vortexed. After centrifugation at the above parameters, phages present in the supernatant were collected. The transduction of the investigated bacterial strain was repeated until saturation in the number of transduced cells (~two or three rounds) was observable. Finally, plasmids from single colonies were sequenced to reveal tail mutations.

### Quantifying replicative phage contamination

*E. coli* cells containing MGP4240 or MGP4240_gp17^V544G^ and pZE21_p15A plasmids were infected with T7Δ(gp11-12-17) phage to package the pZE21_p15A plasmid. The resulting phage particles were used to generate phage lysates in *E. coli* BW25113 and *S. sonnei* HNCMB 25021 harbouring either MGP4240 or MGP4240_gp17^V544G^. The presence of the phage-tail-encoding plasmids in the target cells was necessary for replicative phage contamination to form plaques. For the plaque assays, 4 ml top agar was prepared and supplemented with 100 µg ml^−1^ streptomycin (Sigma, S6501-25G) and 400 µl of the overnight cultures. Finally, from each phage stock, 10 µl was dropped onto the top agar in 1–10^10^ times dilutions.

### Site-directed mutagenesis of phage-tail-encoding plasmids

For functional metagenomic library delivery, the mutation identified in the T7 gp17^V544G^ phage tail variant was introduced into plasmid MGP4240^21^ by using whole plasmid amplification with primers carrying the corresponding mutation, followed by DpnI (Thermo Fisher, ER1701) treatment to eliminate the original, methylated template plasmid DNA and subsequent gel electrophoresis, gel extraction and self-ligation. The plasmids were then electroporated into *E. coli* BW25113 cells. Transformants carrying the desired constructs were identified by PCR and validated via sequencing.

### Functional selection of antibiotic resistance

Functional selections for resistance were performed on Mueller Hinton Broth (Sigma, 90922) agar plates containing a concentration gradient of a given antibiotic (adapted from ref. ^54^). Antibiotics were purchased from Sigma or MedChem Express. The number of plated cells covered at least 10× the size of the corresponding metagenomic library. Plates were incubated at 37 °C for 24 h. For each functional selection, a control plate was prepared with the same number of cells containing the empty plasmid (that is, the plasmid without a cloned DNA fragment in the multiple cloning site) that showed the inhibitory zone of the antimicrobial compound for the cells without any resistance plasmid. The resistant clones from the libraries were isolated by washing together the sporadic colonies from the plate region (distal to the inhibition zone and containing higher antibiotic concentration), defined by visual inspection in comparison to the inhibition zone from the control plate. Half of the culture suspended in LB was used for plasmid isolation (GeneJET plasmid miniprep kit; Thermo Fisher, PLN70-1KT), and the rest was frozen with glycerol and stored at −80 °C.

### Sample preparation for sequencing

The obtained resistance-conferring plasmids were sequenced with a hybrid sequencing pipeline (Extended Data Fig. 5) based on ref. ^34^. Long-read sequencing identifies the metagenomic DNA fragments (inserts) and the two 10-nt-long random barcodes pre-cloned up- and down-stream (uptag and downtag, respectively) of each metagenomic DNA fragment. Aliquots of plasmid DNA preparations obtained from each screen were pooled in an equimolar ratio. Genomic DNA contamination was removed from the mixture by Lambda-exonuclease and Exonuclease-I double digestion. The resulting sample was cleaned (DNA Clean and Concentrator-5, Zymo Research kit) and quantified. Next, the plasmid mixture was linearized by adding 5 U of SrfI restriction endonuclease (NEB, R0629S) for every 1 µg of plasmid DNA (one h at 37 °C, followed by inactivation at 65 °C for 20 min), and DNA was quantified using Qubit dsDNA broad-range assay kit (Thermo Fisher,Q33266) before applying to Oxford Nanopore long-read sequencing. Parallel, multiplexed short-read deep sequencing was applied on each functional metagenomic plasmid DNA preparation (previous pooling) to associate nanopore contigs with screening samples (Extended Data Fig. 5). To this end, we amplified the up- and downtag barcodes on the plasmid preparations of each selection experiment separately, using Illumina specific forward and reverse primer pairs. Each primer pair contained P5 and P7 adapter sequences, respectively, and 8-nt-long barcodes for multiplexing and plasmid annealing sites (Supplementary Table 11). We performed PCR using Phusion high-fidelity DNA polymerase (Thermo Fisher, F530S) using the following reaction mixture: 15 ng of template plasmid DNA, 4 µl 5× GC buffer, 0.2 µl Phusion high-fidelity DNA polymerase, 0.6 µl DMSO (dimethyl sulfoxide), 0.2 mM dNTPs, 0.5–0.5 µM forward and reverse primers and water in a final volume of 20 µl. The following thermocycler conditions were used: 95 °C for five min, 30 cycles of 95 °C for 30 s + 59 °C for 30 s + 72 °C for 5 s, 72 °C for seven min. Following concentration measurement of each PCR reaction, we mixed the samples in a 1:1 mass ratio. Next, we isolated the 137-bp-long fragment mixture from 0.75% agarose gel.

### Nanopore sequencing

Libraries were prepared by using a ligation sequencing kit (Oxford Nanopore Technologies, SQK-LSK109) with 1 µg plasmid DNA. The DNA was end-prepped with the NEBNext FFPE Repair (M6630S) and Ultra II End Prep kit (E7546S), purified using Agencourt AMPure XP (Beckman Coulter, A63882) and then the adapter ligated using NEBNext Quick T4 DNA ligase (E6056S). Finally, the adapted library was purified by Agencourt AMPure, quantified using Qubit 3.0, mixed with ONT running buffer and loading beads, primed with FLO-MIN106 9.4.1 SpotON flow cell attached to a MinION device and run for 72 h. Guppy algorithm (v8.25) with high-accuracy config settings was used for basecalling. Raw reads were filtered on the basis of quality value (QC ≥ 7) and length (4,000–8,000 bp) using NanoFilt v2.7.1^55^. Reads were mapped to the reference sequence with minimap2 (v2.17)^56^; SAM files were converted to sorted BAMs; the insert sequences were exctracted, and barcodes were identified and added to the read/insert names applying samtools tview (1.11-9-ga53817f) subcommand^57^; individual FASTQ files were created using SEQTK (v0.13.2)^58^; consensus sequences were generated using SPOA (v4.0.2)^59^ with the following parameters: -l 0 -r 0 -g -2. Finally, the raw consensus inserts were polished using the relevant set of insert sequences by minimap2 and racon (v1.4.19)^56^ to create the final consensus inserts with at least 100× coverage. Delivered metagenomic DNA fragment lengths and diversities were determined by using long-read deep sequencing right after electroporation into *E. coli* BW25113 and transduction into *Salmonella enterica* subsp. *enterica* serovar *Typhimurium* str. LT2, *K*. *pneumoniae* NCTC 9131 and *S. sonnei* HNCMB 25021. Shannon alpha diversity indices (*H*) were calculated on the basis of the frequency of each of the contigs of all hosts using the vegan R package (2.5-7)^60^.

### Illumina sequencing

Pooled sequencing libraries were denatured with 0.1 M NaOH, diluted to 12 pM with HT1 hybridization buffer (Illumina) and mixed with 40% PhiX Control v3 (Illumina) sequencing control library. Denatured sequencing pools were loaded onto MiSeq Reagent kit V2-300 (Illumina) and 2 × 70 bp sequence reads were generated with an Illumina MiSeq instrument with custom read 1, read 2 and index 1 sequencing primers spiked in the appropriate cartridge positions (12, 14 and 13, respectively) at a final concentration of 0.5 µM.

### Host ranges of the ARGs encoded by the functional metagenomic DNA contigs

Resistant plasmid pools collected from the metagenomic screen were mixed and re-transformed or re-electroporated into the four hosts. Selection experiments were performed on gradient agar plates as described previously (see ‘Functional selection of antibiotic resistance’ above). Resistant colonies were collected and following plasmid preparation, barcodes on the plasmids were sequenced by Illumina sequencing (Supplementary methods). For calculating the overlaps between functional ARG sets across species, we first estimated the accuracy of the screen by comparing the results to that of the MIC measurements of the 13 selected resistance-conferring DNA fragments. On the basis of these comparisons, we estimated the true positive, false positive, true negative and false negative rates of the screen. Next, we calculated an adjusted Jaccard index for each species pair, which takes into account the screen’s accuracy as follows. For each species, we replaced the original vector of presence/absence of detected resistance instances with a new vector where the original presence (absence) values were randomly kept with a probability equal to the positive (negative) predictive value (that is, the proportion of true positives among all positive cases and the proportion of true negatives among all negative cases). The procedure was repeated 50,000 times, and the medians and 95% confidence intervals of the Jaccard indices between pairs of species were calculated.

### Resistance levels in the bacterial hosts

We measured how DNA fragments that provide antibiotic resistance to *E. coli* influence susceptibility in *Shigella sonnei* HNCMB 25021, *K. pneumoniae* NCTC 9131 and *Salmonella enterica* subsp. *enterica* serovar *Typhimurium* str. LT2. For this purpose, we used a representative set of 13 plasmids that were isolated in our antibiotic selection screens. For each strain, the provided resistance levels (that is, the MIC) were measured with a standard 12-step microdilution method in 96-well plates, and the MIC fold change was determined by comparing them to the MIC of the corresponding empty vector harbouring control strains. MICs were determined on the basis of cell growth (OD_600_) after 24 h incubation (37 °C, 180 r.p.m.).

### Sequencing data analysis and functional annotation of ARGs

Each consensus insert sequence from nanopore sequencing was associated with screening samples (host, resistome, antibiotic) by combining the Nanopore and Illumina datasets through the unique uptag and downtag barcodes with a custom R script. To identify ARGs in the metagenomic contigs, two parallel approaches were used: (1) Open Reading Frame (ORF) prediction with prodigal ^61^, followed by annotation with BLASTP search against CARD^35^ and ResFinder^36^ databases, with coverage >50 bp at *e*-value < 10^−5^ and (2) BLASTX search with the same parameters but without ORF prediction to decrease the risk of truncated ORFs due to frame-shifting sequencing errors. To remove low-fidelity sequencing data from the dataset, metagenomic DNA fragments supported by <10 consensus insert sequences in the nanopore dataset and <9 reads in the Illumina uptag and downtag barcode dataset were filtered out.

If a metagenomic DNA fragment contained more than one predicted ARG, ARGs known to act on an antibiotic class (based on CARD and ResFinder reference databases) other than the one we used in the selection experiment were filtered out. ARG sequences having at least 95% identity and coverage on the DNA sequence level were collapsed into ARG clusters^37^. Each cluster was represented by the closest hit to known ARGs in the Card^35^ and ResFinder^36^ databases (Supplementary Table 6). Donor organisms from which the assembled DNA contig sequences originated were identified by nucleotide sequence similarity search using the DNA contigs as query against the NCBI Reference Prokaryotic database (RefProk, downloaded 21 March 2021) with a threshold *e*-value of 10^−10^. The taxonomic hierarchy (kingdom, phylum, class, order, family, genus, species) was acquired using the taxonomizr package in R (v0.8.0).

### Mobilization of the isolated ARGs

To create the mobile gene catalogue (that is, a database of recently transferred DNA sequences between bacterial species^40^), we downloaded 1,377 genomes of diverse human-related bacterial species from the Integrated Microbial Genomes and Microbiomes database as done previously^40^ and 1,417 genomes of Gram-negative ESKAPE pathogens from the NCBI RefSeq database (Supplementary Table 8). Using NCBI blastn 2.10.1+^62^, we searched the nucleotide sequences shared between genomes belonging to different species. The parameters for filtering the NCBI blastn 2.10.1+ blast results were the following: minimum percentage of identity, 99%; minimum alignment length, 500; maximum alignment length, 20,000. The blast hits were clustered by cd-hit-est 4.8.1^63,64^, with sequence identity threshold of 99%. We predicted the ORFs on the blast hits with prodigal v2.6.3^61^, keeping only those longer than 500 nt. Then, to generate the mobile gene catalogue, we compared them with the merged CARD 3.1.0^35^ and ResFinder (d48a0fe)^36^ databases using diamond v2.0.4.142^65^. Finally, natural plasmid sequences were identified by downloading 27,939 complete plasmid sequences from the PLSDB database (v2020-11-19)^41^. Then, representative sequences of the isolated 114 ARG clusters were BLASTN searched both in the mobile gene catalogue and in natural plasmid sequences, with an identity and coverage threshold of 90%. ARGs that were present in the mobile gene catalogue and/or in natural plasmid sequences were considered as mobile.

### Statistical analysis

Statistical analysis was performed using R (v4.1.1). The parametric two-sample *t*-test was used to assess the differences between the means of the groups of samples. Fisher’s exact test was used to determine significant associations between two variables. Shannon alpha diversity index was used to characterize the diversity of DNA contigs in the libraries using the vegan package (v2.5–7) in R^66^. Data distribution was assumed to be normal, but this was not formally tested.

### Reporting summary

Further information on research design is available in the Nature Portfolio Reporting Summary linked to this article.

### Supplementary information

Supplementary Information Supplementary methods, Description of Extended Data Table 1, Supplementary Tables 1–11 and Source Data Extended Data Figs. 1–4.

Reporting Summary.

Supplementary Table Supplementary Tables 1–11.

### Source data

Source Data Extended Data Fig. 1 Uncropped scan of gel picture in Extended Data Fig. 1 (Klebsiella *pneumoniae* NCTC 9131 + Soil library by K11 phage).

Source Data Extended Data Fig. 2 Uncropped scan of gel picture in Extended Data Fig. 2 (*Salmonella enterica* subsp. *enterica* serovar *Typhimurium* str. LT2 + Gut library by ΦSG-JL2 phage).

Source Data Extended Data Fig. 3 Uncropped scan of gel picture in Extended Data Fig. 3 (*Salmonella enterica* subsp. *enterica* serovar *Typhimurium* str. LT2 + Clinical library by ΦSG-JL2 phage).

Source Data Extended Data Fig. 4 Uncropped scan of gel picture in Extended Data Fig. 4 (Electroporation into *Escherichia coli* K12 BW25113).

## Figures and Tables

**Extended Data Table 1:**
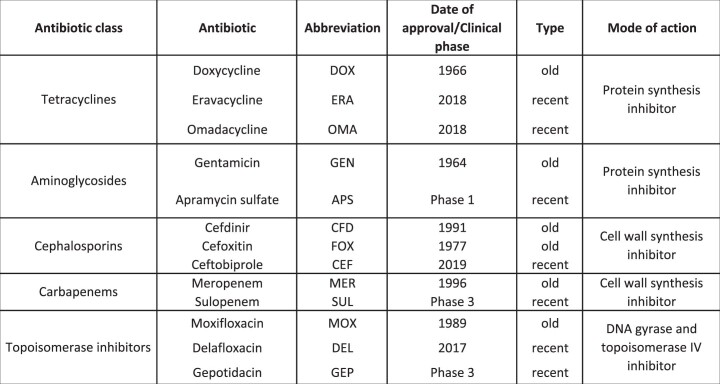
List of antibiotics used in this study

Seven recent antibiotics were selected based on WHO reports (https://www.who.int/publications/i/item/9789240021303) and commercial availability.

## Data Availability

Illumina reads and Nanopore contigs for this study have been deposited in the European Nucleotide Archive (ENA) at EMBL-EBI under accession number PRJEB54063 (https://www.ebi.ac.uk/ena/browser/view/PRJEB54063). Source data are provided with this paper.

## Code availability

Scripts and other files needed to reproduce the analysis are available at https://github.com/stitam/Apjok-et-al-DEEPMINE-NatMicrobiol.

## References

[1] Tringe SG, Rubin EM (2005). Metagenomics: DNA sequencing of environmental samples. Nat. Rev. Genet..

[2] Coughlan LM, Cotter PD, Hill C, Alvarez-Ordóñez A (2015). Biotechnological applications of functional metagenomics in the food and pharmaceutical industries. Front. Microbiol..

[3] Daniel R (2005). The metagenomics of soil. Nat. Rev. Microbiol..

[4] Lorenz P, Eck J (2005). Metagenomics and industrial applications. Nat Rev Microbiol.

[5] Colin P-Y (2015). Ultrahigh-throughput discovery of promiscuous enzymes by picodroplet functional metagenomics. Nat. Commun..

[6] Crofts TS, Gasparrini AJ, Dantas G (2017). Next-generation approaches to understand and combat the antibiotic resistome. Nat. Rev. Microbiol..

[7] Forsberg KJ (2019). Functional metagenomics-guided discovery of potent cas9 inhibitors in the human microbiome. eLife.

[8] van der Helm E, Genee HJ, Sommer MOA (2018). The evolving interface between synthetic biology and functional metagenomics. Nat. Chem. Biol..

[9] Boolchandani, M., Patel, S. & Dantas, G. Functional metagenomics to study antibiotic resistance. In Sass, P. (ed) *Antibiotics. Methods in molecular biology*, vol 1520, 307-329 (Humana Press, New York, 2017). 10.1007/978-1-4939-6634-9_19 27873261

[10] dos Santos DFK, Istvan P, Quirino BF, Kruger RH (2017). Functional metagenomics as a tool for identification of new antibiotic resistance genes from natural environments. Microb. Ecol..

[11] Lam KN, Martens EC, Charles TC (2018). Developing a Bacteroides system for function-based screening of DNA from the human gut microbiome. mSystems.

[12] Taupp M, Mewis K, Hallam SJ (2011). The art and design of functional metagenomic screens. Curr. Opin. Biotechnol..

[13] Ngara TR, Zhang H (2018). Recent advances in function-based metagenomic screening. Genom. Proteom. Bioinform..

[14] Uchiyama T, Miyazaki K (2009). Functional metagenomics for enzyme discovery: challenges to efficient screening. Curr Opin Biotechnol.

[15] Lammens EM, Nikel PI, Lavigne R (2020). Exploring the synthetic biology potential of bacteriophages for engineering non-model bacteria. Nat Commun..

[16] Sommer MOA, Church GM, Dantas G (2010). The human microbiome harbors a diverse reservoir of antibiotic resistance genes. Virulence.

[17] Pehrsson EC (2016). Interconnected microbiomes and resistomes in low-income human habitats. Nature.

[18] Sommer MOA, Dantas G, Church GM (2009). Functional characterization of the antibiotic resistance reservoir in the human microflora. Science.

[19] Apjok G (2019). Limited evolutionary conservation of the phenotypic effects of antibiotic resistance mutations. Mol. Biol. Evol..

[20] Porse A, Schou TS, Munck C, Ellabaan MMH, Sommer MOA (2018). Biochemical mechanisms determine the functional compatibility of heterologous genes. Nat Commun..

[21] Yosef I, Goren MG, Globus R, Molshanski-Mor S, Qimron U (2017). Extending the host range of bacteriophage particles for DNA transduction. Mol. Cell.

[22] Nyerges Á (2018). Directed evolution of multiple genomic loci allows the prediction of antibiotic resistance. Proc. Natl Acad. Sci. USA.

[23] Wright GD (2019). Environmental and clinical antibiotic resistomes, same only different. Curr. Opin. Microbiol..

[24] Bengtsson-Palme J, Boulund F, Fick J, Kristiansson E, Joakim Larsson DG (2014). Shotgun metagenomics reveals a wide array of antibiotic resistance genes and mobile elements in a polluted lake in India. Front. Microbiol..

[25] Lübbert C (2017). Environmental pollution with antimicrobial agents from bulk drug manufacturing industries in Hyderabad, South India, is associated with dissemination of extended-spectrum beta-lactamase and carbapenemase-producing pathogens. Infection.

[26] Bakermans C, Sloup RE, Zarka DG, Tiedje JM, Thomashow MF (2009). Development and use of genetic system to identify genes required for efficient low-temperature growth of *Psychrobacter arcticus* 273-4. Extremophiles.

[27] Tridgett M, Ababi M, Osgerby A, Garcia RR, Jaramillo A (2020). Engineering bacteria to produce pure phage-like particles for gene delivery. ACS Synth. Biol..

[28] Huss P, Meger A, Leander M, Nishikawa K, Raman S (2021). Mapping the functional landscape of the receptor binding domain of t7 bacteriophage by deep mutational scanning. eLife.

[29] Yehl K (2019). Engineering phage host-range and suppressing bacterial resistance through phage tail fiber mutagenesis. Cell.

[30] Holtzman T, Globus R, Molshanski-Mor S, Ben-Shem A, Yosef I, Qimron U (2020). A continuous evolution system for contracting the host range of bacteriophage T7. Sci Rep..

[31] Qimron U, Marintcheva B, Tabor S, Richardson CC (2006). Genomewide screens for *Escherichia coli* genes affecting growth of T7 bacteriophage. Proc. Natl Acad. Sci. USA.

[32] Lupia T, Pallotto C, Corcione S, Boglione L, De Rosa FG (2021). Ceftobiprole perspective: current and potential future indications. Antibiotics.

[33] Sou T (2021). Model‐informed drug development for antimicrobials: translational PK and PK/PD modeling to predict an efficacious human dose for apramycin. Clin. Pharmacol. Ther..

[34] Mutalik VK (2019). Dual-barcoded shotgun expression library sequencing for high-throughput characterization of functional traits in bacteria. Nat. Commun..

[35] Alcock BP (2020). CARD 2020: antibiotic resistome surveillance with the comprehensive antibiotic resistance database. Nucleic Acids Res..

[36] Zankari E (2012). Identification of acquired antimicrobial resistance genes. J. Antimicrob. Chemother..

[37] Ellabaan MMH, Munck C, Porse A, Imamovic L, Sommer MOA (2021). Forecasting the dissemination of antibiotic resistance genes across bacterial genomes. Nat Commun..

[38] Zhang AN (2021). An omics-based framework for assessing the health risk of antimicrobial resistance genes. Nat Commun..

[39] Hu Y (2016). The bacterial mobile resistome transfer network connecting the animal and human microbiomes. Appl. Environ. Microbiol..

[40] Smillie CS (2011). Ecology drives a global network of gene exchange connecting the human microbiome. Nature.

[41] Galata V, Fehlmann T, Backes C, Keller A (2019). PLSDB: a resource of complete bacterial plasmids. Nucleic Acids Res..

[42] Cillóniz C, Dominedò C, Garcia-Vidal C, Torres A (2019). Ceftobiprole for the treatment of pneumonia. Rev. Esp. Quimioter.

[43] Torres A, Liapikou A, Cilloniz C (2015). Ceftobiprole for the treatment of pneumonia: a European perspective. Drug Des. Dev. Ther.

[44] Queenan AM, Shang W, Kania M, Page MGP, Bush K (2007). Interactions of ceftobiprole with β-lactamases from molecular classes A to D. Antimicrob. Agents Chemother..

[45] Farrell DJ, Flamm RK, Sader HS, Jones RN (2014). Ceftobiprole activity against over 60,000 clinical bacterial pathogens isolated in Europe, Turkey, and Israel from 2005 to 2010. Antimicrob. Agents Chemother..

[46] Hao M (2021). Apramycin resistance in epidemic carbapenem-resistant *Klebsiella pneumoniae* ST258 strains. J. Antimicrob. Chemother..

[47] Tasse L (2010). Functional metagenomics to mine the human gut microbiome for dietary fiber catabolic enzymes. Genome Res..

[48] Kakirde KS (2011). Gram negative shuttle BAC vector for heterologous expression of metagenomic libraries. Gene.

[49] Rousset F (2021). The impact of genetic diversity on gene essentiality within the *Escherichia coli* species. Nat. Microbiol..

[50] MacLean RC, San Millan A (2019). The evolution of antibiotic resistance. Science.

[51] Amos GCA, Zhang L, Hawkey PM, Gaze WH, Wellington EM (2014). Functional metagenomic analysis reveals rivers are a reservoir for diverse antibiotic resistance genes. Vet. Microbiol..

[52] Cheng G (2012). Functional screening of antibiotic resistance genes from human gut microbiota reveals a novel gene fusion. FEMS Microbiol. Lett..

[53] Fick J (2009). Contamination of surface, ground, and drinking water from pharmaceutical production. Environ. Toxicol. Chem..

[54] Soper, W. T. Modified gradient plate for use in the virus plaque technique. *Appl. Microbiol.*10.1128/am.14.3.470-471.1966 (1966). 10.1128/am.14.3.470-471.1966 PMC546748 16349664

[55] De Coster W, D’Hert S, Schultz DT, Cruts M, Van Broeckhoven C (2018). NanoPack: visualizing and processing long-read sequencing data. Bioinformatics.

[56] Li H (2018). Minimap2: pairwise alignment for nucleotide sequences. Bioinformatics.

[57] Li H (2009). The Sequence Alignment/Map format and SAMtools. Bioinformatics.

[58] Li, H. Seqtk: A fast and lightweight tool for processing FASTA or FASTQ Sequences. github. https://github.com/lh3/seqtk (2013).

[59] Vaser R, Sović I, Nagarajan N, Šikić M (2017). Fast and accurate de novo genome assembly from long uncorrected reads. Genome Res..

[60] Fisher, R. A., Corbet, A. S. & Williams, C. B. The relation between the number of species and the number of individuals in a random sample of an animal population. *J. Anim. Ecol.* **12**, 42-58 (1943).

[61] Hyatt D (2010). Prodigal: prokaryotic gene recognition and translation initiation site identification. BMC Bioinformatics.

[62] Altschul SF, Gish W, Miller W, Myers EW, Lipman DJ (1990). Basic local alignment search tool. J. Mol. Biol..

[63] Fu L, Niu B, Zhu Z, Wu S, Li W (2012). CD-HIT: accelerated for clustering the next-generation sequencing data. Bioinformatics.

[64] Li W, Godzik A (2006). Cd-hit: a fast program for clustering and comparing large sets of protein or nucleotide sequences. Bioinformatics.

[65] Buchfink B, Xie C, Huson DH (2014). Fast and sensitive protein alignment using DIAMOND. Nat. Methods.

[66] Oksanen, J. et al. vegan: Community Ecology Package. R package version 2.6-4 (2022).

