# Supplementary material for: Characterization of antibiotic resistomes by reprogrammed bacteriophage-enabled functional metagenomics in clinical strains
Source: Nat Microbiol. 2023 Feb 9;8(3):410–23. doi: 10.1038/s41564-023-01320-2 (PMC9981461; doi:10.1038/s41564-023-01320-2)
Supplement: Supplementary file 1 — Supplementary methods, Description of Extended Data Table 1, Supplementary Tables 1–11 and Source Data Extended Data Figs. 1–4. [file 41564_2023_1320_MOESM1_ESM.pdf]

nature microbiology

Article

https://doi.org/10.1038/s41564-023-01320-2

Characterization of antibiotic resistomes by reprogrammed bacteriophage-enabled functional metagenomics in clinical strains

In the format provided by the authors and unedited

Supplementary Information

Extended Data Files

Extended Data Table 1: List of antibiotics used in this study. Seven recent antibiotics were selected based on WHO reports (https://www.who.int/publications/i/item/9789240021303) and commercial availability.

Supplementary Tables

Supplementary Table 1: Sources of the metagenomics libraries
Supplementary Table 2: Library sizes acheieved by electroporation and transduction
Supplementary Table 3: Read lengths and diversities of the contigs in the three libraries
Supplementary Table 4: Position of potential HRDR regions of the tail fibers and transduction efficiencies of phages with mutagenised tail fibers
Supplementary Table 5: Transduction efficiencies and plaque formation in *S. sonnei* and *E. coli* by the wild type and mutant versions of T7 phage
Supplementary Table 6: List of contigs identified in the functional selection screens
Supplementary Table 7: List of ARG clusters identified in the functional selection screens
Supplementary Table 8: Mobile gene pool Tablebase
Supplementary Table 9: MIC fold changes provided by the 20 selected contigs
Supplementary Table 10: List of ARG clusters identified in the functional selection screens of the pre-selected plasmid libraries
Supplementary Table 11: List of plasmids and primers used in the study

Source Data Extended Data Figures

Source Data Extended Data Figure 1: Uncropped scan of gel picture on Extended Data Figure 1 (*Klebsiella pneumoniae* NCTC 9131 + Soil library by K11 phage)
Source Data Extended Data Figure 2**.** Uncropped scan of gel picture on Extended Data Figure 1 (*Salmonella enterica* subsp. enterica serovar Typhimurium str. LT2 + Gut library by ΦSG-JL2 phage)
Source Data Extended Data Figure 3**.** Uncropped scan of gel picture on Extended Data Figure 1 (*Salmonella enterica* subsp. enterica serovar Typhimurium str. LT2 + Clinical library by ΦSG-JL2 phage)
Source Data Extended Data Figure 4**.** Uncropped scan of gel picture on Extended Data Figure 1 (Electroporation into *Escherichia coli* K12 BW25113)
