# Supplementary material for: Characterization of antibiotic resistomes by reprogrammed bacteriophage-enabled functional metagenomics in clinical strains
Source: Nat Microbiol. 2023 Feb 9;8(3):410–23. doi: 10.1038/s41564-023-01320-2 (PMC9981461; doi:10.1038/s41564-023-01320-2)
Supplement: Supplementary file 2 — Reporting Summary. [file 41564_2023_1320_MOESM2_ESM.pdf]

nature portfolio

Corresponding author(s): Balint Kintses, Csaba Pál

Last updated by author(s): Dec 27, 2022

Reporting Summary

Nature Portfolio wishes to improve the reproducibility of the work that we publish. This form provides structure for consistency and transparency in reporting. For further information on Nature Portfolio policies, see our Editorial Policies and the Editorial Policy Checklist.

Statistics

For all statistical analyses, confirm that the following items are present in the figure legend, table legend, main text, or Methods section.

n/a	Confirmed	
☐	☒	The exact sample size (*n*) for each experimental group/condition, given as a discrete number and unit of measurement
☐	☒	A statement on whether measurements were taken from distinct samples or whether the same sample was measured repeatedly
☐	☒	The statistical test(s) used AND whether they are one- or two-sided<br>*Only common tests should be described solely by name; describe more complex techniques in the Methods section.*
☐	☒	A description of all covariates tested
☐	☒	A description of any assumptions or corrections, such as tests of normality and adjustment for multiple comparisons
☐	☒	A full description of the statistical parameters including central tendency (e.g. means) or other basic estimates (e.g. regression coefficient) AND variation (e.g. standard deviation) or associated estimates of uncertainty (e.g. confidence intervals)
☐	☒	For null hypothesis testing, the test statistic (e.g. *F*, *t*, *r*) with confidence intervals, effect sizes, degrees of freedom and *P* value noted<br>*Give P values as exact values whenever suitable.*
☒	☐	For Bayesian analysis, information on the choice of priors and Markov chain Monte Carlo settings
☒	☐	For hierarchical and complex designs, identification of the appropriate level for tests and full reporting of outcomes
☒	☐	Estimates of effect sizes (e.g. Cohen's *d*, Pearson's *r*), indicating how they were calculated

Our web collection on statistics for biologists contains articles on many of the points above.

Software and code

Policy information about availability of computer code

Data collection	We did not use any code or software to collect data for this study.
Data analysis	Guppy algorithm (v8.25), NanoFilt v 2.7.1. , SEQTK (v0.13.2), SPOA (v4.0.2), minimap2 (ver 2.17), samtools tview (1.11-9-ga53817f), racon (v1.4.19), prodigal v2.6.3, diamond v2.0.4.142, CARD 3.1.0, ResFinder (d48a0fe), NCBI blastn 2.10.1+, cd-hit-est 4.8.1, RefProk (03.21.2021), taxonomizr R package (v0.8.0), vegan R package (2.5-7), PLSDB database (version 2020-11-19)

For manuscripts utilizing custom algorithms or software that are central to the research but not yet described in published literature, software must be made available to editors and reviewers. We strongly encourage code deposition in a community repository (e.g. GitHub). See the Nature Portfolio guidelines for submitting code & software for further information.

Data

Policy information about availability of data

All manuscripts must include a data availability statement. This statement should provide the following information, where applicable:

- Accession codes, unique identifiers, or web links for publicly available datasets
- A description of any restrictions on data availability
- For clinical datasets or third party data, please ensure that the statement adheres to our policy

Illumina reads and Nanopore contigs for this study have been deposited in the European Nucleotide Archive (ENA) at EMBL-EBI under accession number PRJEB54063 (https://www.ebi.ac.uk/ena/browser/view/PRJEB54063). Scripts and other files needed to reproduce the analysis are available at https://github.com/stitam/Apjok-et-al-DEEPMINE-NatMicrobiol.

Field-specific reporting

Please select the one below that is the best fit for your research. If you are not sure, read the appropriate sections before making your selection.

☒ Life sciences ☐ Behavioural & social sciences ☐ Ecological, evolutionary & environmental sciences

For a reference copy of the document with all sections, see nature.com/documents/nr-reporting-summary-flat.pdf

Life sciences study design

All studies must disclose on these points even when the disclosure is negative.

Sample size	In functional metagenomics the number of tested treatments (13 antibiotics), library sizes (2-6 million clones per environments) and number of DNA sources (10 individuals, 6 polluted sites and 68 bacterial species) met with the standards of previously published studies in the field of antibiotic resistance research (DOI:10.1126/science.1176950, DOI:10.1038/nature13377). The studied 6 old antibiotics served as positive controls for the screens with 7 new antibiotics. The large number of resistant DNA fragments obtained with the antibiotic confirmed that our sample size was large enough for the presented study.
Data exclusions	No data were excluded.
Replication	All experiments were carried out at least in two replicates.
Randomization	Samples were not allocated to experimental groups. For one type of experiments all samples were handled by one person in charge.
Blinding	Data collection and analysis were not performed blind to the conditions of the experiments, because it is not a standard procedure in this field.

Reporting for specific materials, systems and methods

We require information from authors about some types of materials, experimental systems and methods used in many studies. Here, indicate whether each material, system or method listed is relevant to your study. If you are not sure if a list item applies to your research, read the appropriate section before selecting a response.

Materials & experimental systems

n/a	Involved in the study	
☒	☐	Antibodies
☒	☐	Eukaryotic cell lines
☒	☐	Palaeontology and archaeology
☒	☐	Animals and other organisms
☐	☒	Human research participants
☒	☐	Clinical data
☒	☐	Dual use research of concern

Methods

n/a	Involved in the study	
☒	☐	ChIP-seq
☒	☐	Flow cytometry
☒	☐	MRI-based neuroimaging

Human research participants

Policy information about studies involving human research participants

Population characteristics	Human stool samples were obtained from ten healthy unrelated individuals, The five male and 5 female participants were in the age range of 26-42 years.
Recruitment	Volunteer participants were selected based on the strict criteria that (1) they did not take any antibiotics at least for one year prior sample donation, (2) they are in a good health condition. These requirements are standard in the field and secure a bias free comparison of the antibiotic resistomes in the healthy human gut microbiome. Informed consent was obtained by all participants.
Ethics oversight	Human Investigation Review Board of Albert Szent-Györgyi Clinical Centre, University of Szeged (registered under 72/2019-SZTE)

Note that full information on the approval of the study protocol must also be provided in the manuscript.
