# Supplementary figures and images for: Characterization of antibiotic resistomes by reprogrammed bacteriophage-enabled functional metagenomics in clinical strains
Source: Nat Microbiol. 2023 Feb 9;8(3):410–23. doi: 10.1038/s41564-023-01320-2 (PMC9981461; doi:10.1038/s41564-023-01320-2)

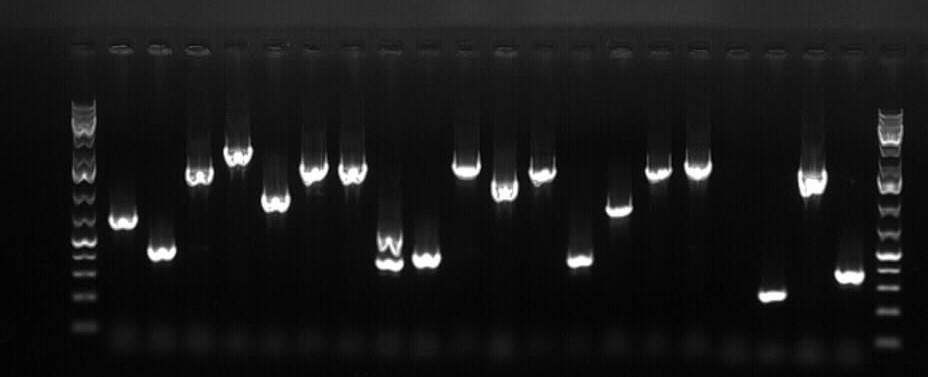

Supplement: Supplementary file 4 — Uncropped scan of gel picture in Extended Data Fig. 1 (Klebsiella pneumoniae NCTC 9131 + Soil library by K11 phage). [file 41564_2023_1320_MOESM4_ESM.jpg]

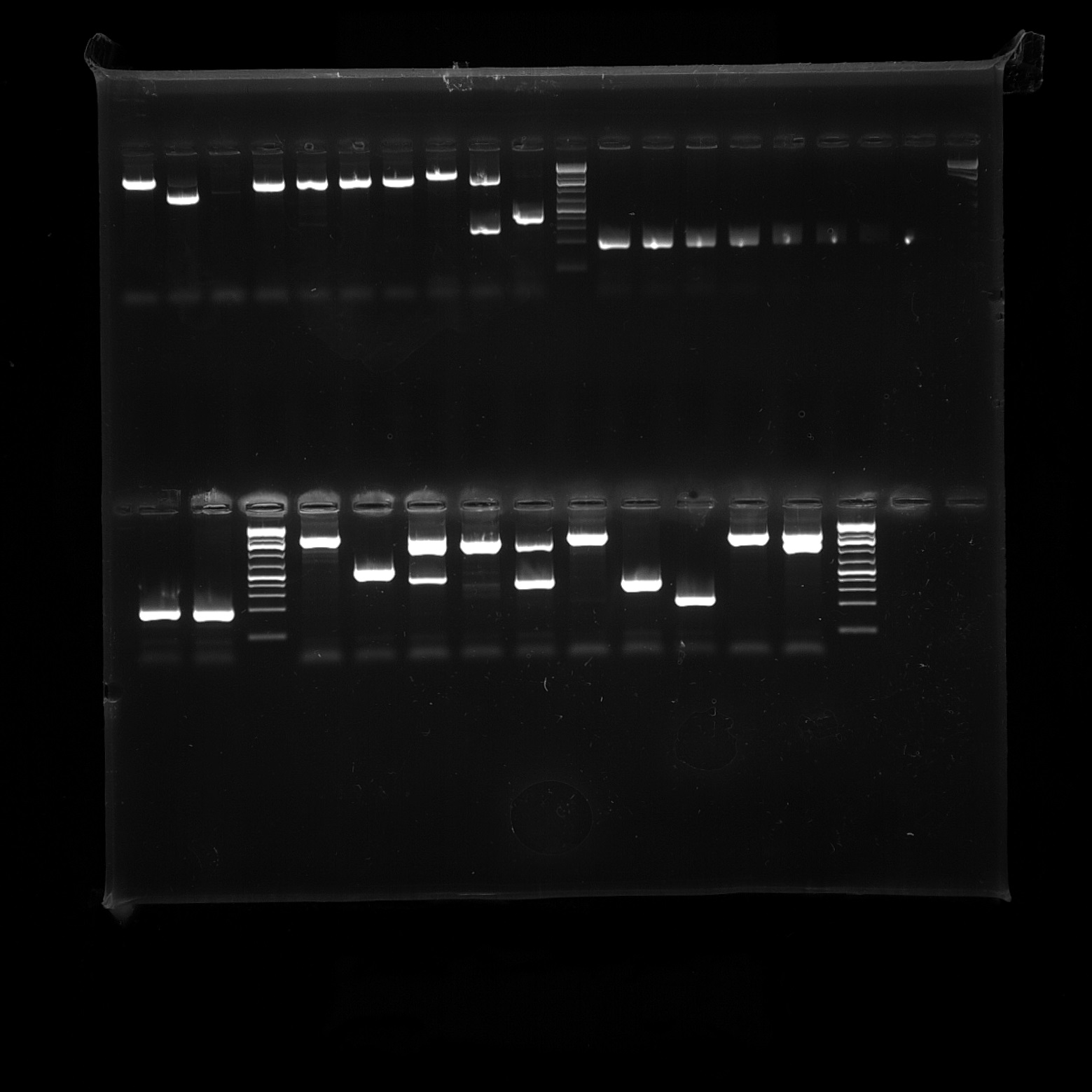

Supplement: Supplementary file 5 — Uncropped scan of gel picture in Extended Data Fig. 2 (Salmonella enterica subsp. enterica serovar Typhimurium str. LT2 + Gut library by ΦSG-JL2 phage). [file 41564_2023_1320_MOESM5_ESM.jpg]

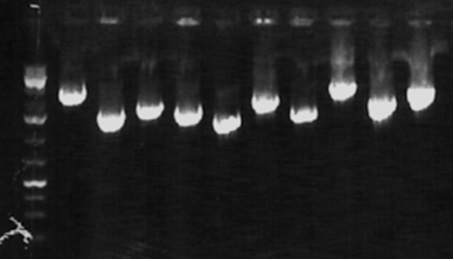

Supplement: Supplementary file 6 — Uncropped scan of gel picture in Extended Data Fig. 3 (Salmonella enterica subsp. enterica serovar Typhimurium str. LT2 + Clinical library by ΦSG-JL2 phage). [file 41564_2023_1320_MOESM6_ESM.jpg]

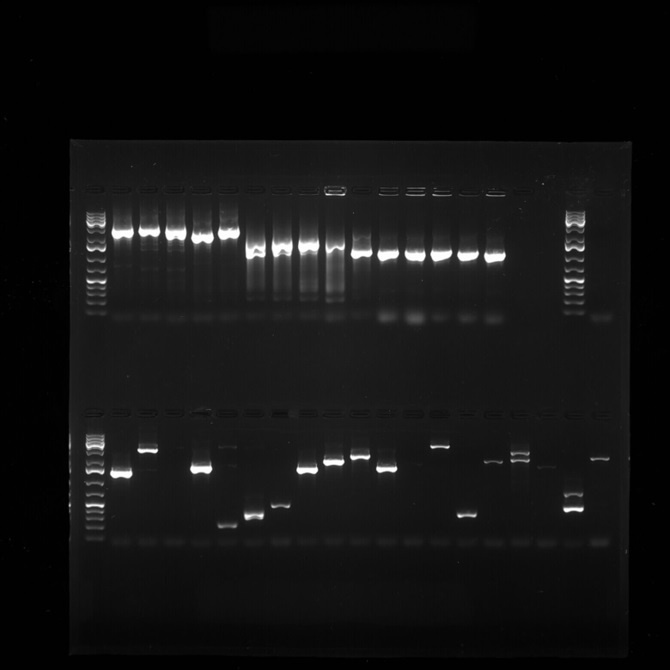

Supplement: Supplementary file 7 — Uncropped scan of gel picture in Extended Data Fig. 4 (Electroporation into Escherichia coli K12 BW25113). [file 41564_2023_1320_MOESM7_ESM.jpg]
